# Complement receptor 1 is expressed on brain cells and in the human brain

**DOI:** 10.1002/glia.24355

**Published:** 2023-02-24

**Authors:** Nikoleta Daskoulidou, Bethany Shaw, Megan Torvell, Lewis Watkins, Emma L. Cope, Sarah M. Carpanini, Nicholas D. Allen, B. Paul Morgan

**Affiliations:** ^1^ UK Dementia Research Institute, Cardiff University Cardiff UK; ^2^ School of Biosciences, Cardiff University Cardiff UK

**Keywords:** Alzheimer's, complement, CR1, microglia

## Abstract

Genome wide association studies (GWAS) have highlighted the importance of the complement cascade in pathogenesis of Alzheimer's disease (AD). Complement receptor 1 (CR1; CD35) is among the top GWAS hits. The long variant of CR1 is associated with increased risk for AD; however, roles of CR1 in brain health and disease are poorly understood. A critical confounder is that brain expression of CR1 is controversial; failure to demonstrate brain expression has provoked the suggestion that peripherally expressed CR1 influences AD risk. We took a multi‐pronged approach to establish whether CR1 is expressed in brain. Expression of CR1 at the protein and mRNA level was assessed in human microglial lines, induced pluripotent stem cell (iPSC)‐derived microglia from two sources and brain tissue from AD and control donors. CR1 protein was detected in microglial lines and iPSC‐derived microglia expressing different *CR1* variants when immunostained with a validated panel of CR1‐specific antibodies; cell extracts were positive for CR1 protein and mRNA. CR1 protein was detected in control and AD brains, co‐localizing with astrocytes and microglia, and expression was significantly increased in AD compared to controls. CR1 mRNA expression was detected in all AD and control brain samples tested; expression was significantly increased in AD. The data unequivocally demonstrate that the CR1 transcript and protein are expressed in human microglia ex vivo and on microglia and astrocytes in situ in the human brain; the findings support the hypothesis that *CR1* variants affect AD risk by directly impacting glial functions.

## Main Points

- CR1 mRNA and protein are expressed in human microglial cell lines.
- iPSC‐derived microglia from CR1 risk and non‐risk donors abundantly express CR1.
- CR1 is expressed in astrocytes and microglia in the human brain and is upregulated in AD.

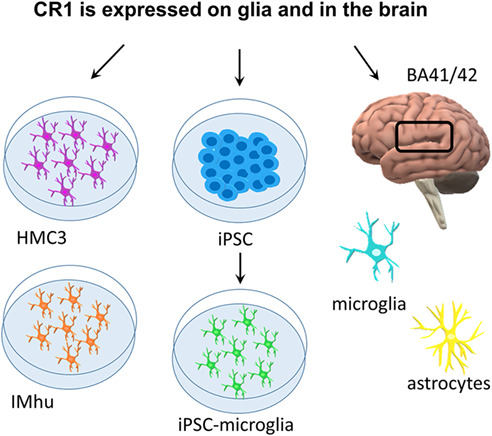

**Abbreviations**
ACM: astrocyte‐conditioned medium
AD: Alzheimer's disease
ADF: advanced DMEM/F12 DMEM
APC: allophycocyanin
APOE: apolipoprotein E
BSA: bovine serum albumin
CR1: complement receptor 1
CR3: complement receptor 3
CytoD: cytochalasin D
EB: embryoid body
FI: factor I
fov: field of view
GM: gray matter
GWAS: genome wide association studies
iPSC: induced pluripotent stem cell
KO: knocked out
LHR: long homologous repeats
mAb: monoclonal antibody
MAC: membrane attack complex
MPC: myeloid precursor cells
NH_2_
: amino terminus
pAb: polyclonal antibody
PBMC: peripheral blood mononuclear cells
PBS: phosphate‐buffered saline
PBST: PBS 0.1% Tween 20
qRT‐PCR: quantitative real‐time PCR
RBC: red blood cells
RFLP: restriction fragment length polymorphism
ROI: regions of interest
SCR: short consensus repeats
sCR1: soluble CR1
SeV: Sendai virus
TM: transmembrane segment

## 1 INTRODUCTION

Complement plays important roles in protection against pathogens and removal of debris in resolution of injury (Ricklin & Lambris, 2013). Dysregulation of complement causes uncontrolled inflammation, tissue damage, and degeneration, which is apparent in many disease contexts. In Alzheimer's disease (AD), complement activation associated with inflammation and glial cell activation has long been recognized but considered secondary to amyloid‐β (Aβ)‐ and tau‐mediated pathology (Musiek & Holtzman, 2015). Recent genetic studies implicate complement as a primary player in AD. *CR1*, encoding complement receptor 1 (CR1/CD35) (Harold et al., 2009; Lambert et al., 2009) and *CLU*, encoding the plasma complement inhibitor clusterin (Lambert et al., 2009), are top GWAS hits in AD, while C*1S*, encoding the complement enzyme C1s, was recently implicated in AD GWAS (Bellenguez et al., 2022). Other GWAS hits include *CD33* and *TREM2*, both of which encode myeloid cell receptors that bind complement proteins C1q and/or C3 (Guerreiro et al., 2013; Naj et al., 2011). Precisely how complement gene variants influence AD risk remains unknown. Knowledge of where the implicated genes are expressed and how they influence brain homeostasis would inform an understanding of pathogenesis and signpost better diagnostic and/or therapeutic tools targeting complement in AD. Here we address the GWAS hit *CR1*.

**FIGURE 1:**
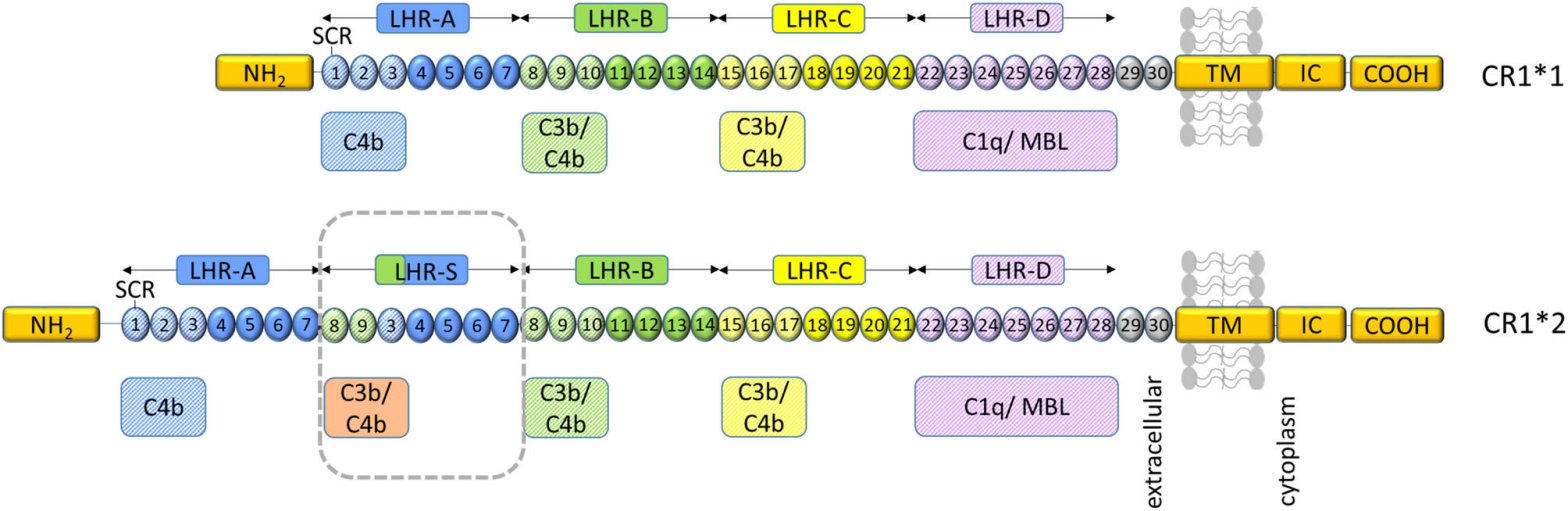
Representation of CR1 structure and ligand binding sites. From the amino terminus (NH_2_) CR1*1 comprises four long homologous repeats (LHRs) each composed of seven short consensus repeats (SCRs) of 60–70 amino acids, two additional SCRs, a transmembrane segment (TM) and an intracytoplasmic carboxy‐terminal domain (IC‐COOH). Each circular block represents an SCR (numbered 1–30). There are three C4b binding sites (SCR 1–3, 8–10, and 15–17) and two C3b binding sites (SCR 8–10 and 15–17). SCRs 22–28 bind C1q, MBL, and ficolins. CR1*2 has an additional LHR domain (LHR‐S) and consequently an extra C3b/C4b binding site. Adapted from Torvell et al. (2021).

CR1 is a membrane receptor that controls complement activation by accelerating decay of the C3 convertase and acting as a co‐factor for factor I (FI)‐mediated conversion of C3b to iC3b, the ligand for the phagocytosis receptor complement receptor 3 (CR3; CD11b/CD18) (Jensen et al., 2021). Soluble CR1 (sCR1) is a disease biomarker in systemic lupus erythematosus (Arora et al., 2004; Hamer et al., 1998) and recombinant sCR1 was developed for therapy with some success in animal models (Carpanini et al., 2019; Morgan & Harris, 2015). The *CR1* gene, located in the “regulators of complement activation” gene cluster on chromosome 1q32, encodes a type 1 transmembrane protein comprising a chain of 60 amino acid repeating units (short consensus repeats; SCR), grouped into sets of seven (long homologous repeats; LHR), each a functional unit with different complement binding properties (Figure 1). Four co‐dominant *CR1* alleles exist, differing in LHR number. The CR1*1 variant (allele frequency 0.87) comprises four LHRs, while the CR1*2 variant (allele frequency 0.11) contains an additional LHR inserted between LHRs A and B, providing an extra C3b/C4b binding site (Figure 1) (Krych‐Goldberg & Atkinson, 2001). CR1*2 is robustly associated with risk for late‐onset AD, faster cognitive decline and greater neuropathological burden (Schmidt et al., 2014; Torvell et al., 2021). CR1*2 carriers have lower levels of brain Aβ, suggesting a role in clearance (Thambisetty et al., 2013), a paradoxical observation prompting the suggestion that Aβ is redistributed into a more neurotoxic form (Gandy et al., 2013).

The cellular and molecular roles of CR1 in the brain and how it influences AD risk are poorly understood. Indeed, brain expression of CR1 remains controversial. A study of brain CR1 expression using western blots, quantitative real‐time PCR (qRT‐PCR), and immunohistochemistry concluded that CR1 was not expressed (Johansson et al., 2018), while others found no CR1 immunoreactivity in normal brain tissue using available reagents (Singhrao et al., 1999). In contrast, CR1 expression on astrocytes was detected in AD and control brains (Fonseca et al., 2016), and CR1 protein and/or mRNA expression has been reported in astrocytes (Gasque et al., 1996) and neurons (Hazrati et al., 2012). A recent study showed CR1 expression in human primary fetal microglia and in microglia differentiated from human induced pluripotent stem cells (iPSC) (Haenseler et al., 2017). Three independent studies reported CR1 mRNA expression in AD cortical homogenates (Allen et al., 2015; Holton et al., 2013; Karch et al., 2012). These conflicting studies prompted us to adopt a multi‐pronged approach to test CR1 expression in human microglia in situ and in vitro. The clarification is critical because the presence of CR1 on brain immune cells would support a direct role in brain immune and inflammatory processes that might explain the impact of CR1 on AD pathology.

## 2 MATERIALS AND METHODS

### 2.1 Chemicals

All reagents and tissue culture plastics, except where otherwise stated, were from Fisher Scientific (Loughborough, United Kingdom) or Sigma Aldrich (Gillingham, United Kingdom) and of analytical grade.

### 2.2 Human tissue

EDTA blood samples (5 mL) were collected from consented healthy donors and anonymized. Erythrocyte membranes were isolated by standard methods (Zelek et al., 2019). Frozen post‐mortem brain tissue from five AD cases (Braak VI) and five age and sex‐matched controls were used in this study (Table S1). The primary auditory cortex (BA41/42) was chosen as a well‐described site of early AD‐associated pathology with strong pathology burden at end stage disease (Jackson et al., 2019).

### 2.3 Cell culture

Human microglial cell lines, HMC3 (ATCC® CRL‐3304™) and Immortalized Human Microglia‐SV40 line (IMhu; Applied Biological Materials, Richmond, BC, Canada), were both derived from embryonic microglia by SV40‐transformation (Chiavari et al., 2019; Dello Russo et al., 2018). The human neuroblastoma cell line, SH‐SY5Y (ATCC® CRL‐2266™), was used as negative control and the human monocytic cell line, THP‐1 (ATCC® TIB‐202™), as positive control. HMC3 and SH‐SY5Y were cultured in Dulbecco's Modified Eagle's Medium (DMEM, ThermoFisher), IMhu in Prigrow III medium (NBS Biologicals) and THP‐1 in RPMI‐1640 medium (ThermoFisher), each supplemented with 10% fetal bovine serum, 100 units/mL penicillin and 100 μg/mL streptomycin (Sigma). IMhu cells were grown in collagen‐coated (Sigma) flasks. KOLF2 (http://www.hipsci.org/lines/#/lines/HPSI0114i-kolf_2) and donor‐derived iPSC were cultured in mTeSR medium (STEMCELL Technologies) on plates coated with Geltrex™ LDEV‐free basement membrane matrix (ThermoFisher). All cells were maintained at 37°C, 95% air and 5% CO_2_, and seeded on polylysine coated coverslips.

### 2.4 *CR1* junction fragment analysis

Genomic DNA was extracted from EDTA blood using the E.Z.N.A.® Tissue DNA Kit (Omega Bio‐tek); PCR products were amplified from 100 ng genomic DNA in a final volume of 25 μL, with 12.5 μL 2× GoTaq Green Master Mix, and 1 μL of each PCR primer at 10 μM. The PCR primers were 5′‐AAT GTG TTT TGA TTT CCC AAG ATC AG‐3′ (forward) and 5′‐CTC AAC CTC CCA AAG GTG CTA‐3′ (reverse). A touch‐down PCR protocol was used (Kucukkilic et al., 2018), with an initial denaturation step of 95°C for 2 min, followed by 20 cycles of 95°C for 30 s, 70°C for 30 s decreasing by 0.5°C every cycle to 60°C, and 70°C for 30 s. These 20 cycles were then followed by 15 cycles of 95°C for 30 s, 60°C for 30 s and 70°C for 30 s, then a final extension step of 70°C for 5 min. PCR products were analyzed on a 1.5% agarose gel stained with SYBR Safe DNA gel stain and visualized under ultraviolet light in a Syngene Gbox.

### 2.5 CR1 density polymorphism genotyping

The density polymorphism of CR1 dictating expression level on erythrocytes was determined by identifying the HindIII restriction fragment length polymorphism (RFLP) using PCR amplification followed by restriction enzyme digestion (Cornillet et al., 1991). PCR products were amplified by incubating genomic DNA (100 ng) in a final volume of 50 μL, with 25 μL 2× GoTaq PCR Master Mix and 1 μL of 10 μM of each PCR primer. The PCR primers used were 5′‐CCT TCA ATG GAA TGG TGC AT‐3′ and 5′‐CCC TTG TAA GGC AAG TCT GG‐3′. The PCR protocol comprised an initial denaturation step of 95°C for 2 min, followed by 30 cycles of 95°C for 30 s, 60°C for 30 s, and 72°C for 1 min, then a final extension step of 72°C for 5 min. To identify the RFLP, 25 μL of PCR product was digested with 100 units of HindIII (R0104T, New England Biolabs) for 1 h at 37°C followed by inactivation for 20 min at 80°C. The digested products were then analyzed on a 2% agarose gel along with control samples for each genotype. The gel was stained with SYBR Safe DNA gel stain and visualized under ultraviolet light in a Syngene Gbox. HindIII digestion did not alter the PCR product (1.8 kb) from individuals who were homozygous for the CR1 high‐density allele (HH), whereas the PCR product was fully cleaved to 1.3 and 0.5 kb bands in samples from individuals homozygous for the CR1 low‐density allele (LL).

### 2.6 Apolipoprotein E genotyping

Apolipoprotein E (*APOE*) status was determined as previously described (Ingelsson et al., 2003). PCR products were amplified from 500 ng genomic DNA in a final volume of 50 μL, with 25 μL 2× GoTaq PCR Master Mix, and 2 μL of 10 μM of each PCR primer. The PCR primers used were 5′‐TAA GCT TGG CAC GGC TGT CCA AGG A‐3′ (forward) and 5′‐ACA GAA TTC GCC CCG GCC TGG TAC ACT GCC‐3′ (reverse). The PCR protocol comprised an initial denaturation step of 95°C for 10 min, followed by 30 cycles of 95°C for 30 s, 56°C for 30 s, and 72°C for 1 min, then a final extension step of 72°C for 5 min. PCR products (25 μL) were digested with 20 units HhaI (R0139S, New England Biolabs) for 1 h at 37°C followed by inactivation for 20 min at 65°C. The digested products were separated on a 4% agarose gel, stained with SYBR Safe DNA gel stain and visualized under ultraviolet light. The band patterns differentiate APOE genotypes as described (Ingelsson et al., 2003).

### 2.7 Reprogramming of PBMC to iPSC expressing CR1*1 or/and CR1*2

Blood samples from homozygote (CR1*1/CR1*1, CR1*2/CR1*2) and heterozygote (CR1*1/CR1*2, CR1*3/CR1*4) donors were collected and peripheral blood mononuclear cells (PBMC) isolated using SepMate™ tubes (STEMCELL Technologies) and Ficoll Paque Plus (GE Healthcare) following the manufacturer's protocol. PBMC were reprogrammed to iPSC using the CytoTune‐iPS 2.0 Sendai Reprogramming Kit (ThermoFisher) following the manufacturer's protocol. Briefly, PBMC were transduced with the CytoTune‐iPS 2.0 Sendai Reprogramming vectors KOS, hc‐Myc, and hKlf4 at multiplicities of infection of 5, 5, and 3 respectively. Cells were incubated overnight and the following day the medium was replaced with fresh complete PBMC medium [StemPro™‐34 SFM supplemented with StemPro™‐34 Nutrient Supplement (STEMCELL Technologies), l‐Glutamine 2 mM, SCF 100 ng/mL, FLT‐3100 ng/mL, IL‐3 20 ng/mL, IL‐6 20 ng/mL] to remove the reprogramming vectors. Two days later, the transduced cells were plated on mitotically arrested mouse embryonic fibroblasts (feeder cells, ThermoFisher) in complete StemPro™‐34 SFM medium without cytokines; medium was replaced after 2 days. Cells were then transitioned into mTeSR iPSC medium (STEMCELL Technologies) by stepwise medium change into complete mTesR medium. Cultures were visually monitored for the emergence of iPSC colonies, which were then picked and transferred onto Geltrex™ LDEV‐free reduced growth factor basement membrane matrix (ThermoFisher)‐coated plates for expansion. iPSC clones were grown, characterized for the expression of pluripotency markers (SOX2, OCT4, TRA‐1‐60, SEEA4) by immunofluorescence and Sendai virus (SeV) clearance, and expanded to produce a cell bank of iPSC lines expressing either CR1*1 or CR1*2 or both. For all lines, copy number variation testing (Illumina global screening array v3.0) was conducted and showed no aneuploidy or other gross chromosomal abnormalities.

### 2.8 Generation of CR1 knockout lines using CRISPR‐Cas9

Paired guide RNAs targeting *CR1* exon 2 were designed using Deskgen (Crisprsystem.com; 5′‐GTC AAT GCA ATG CCC CAG AAT GG‐3′ forward, 5′‐GAG TCA GAC CTG ACC ACG AT‐3′ reverse, Figure S1). Selected guides had one potential off‐target binding site within CR1L. Cas9 enzyme and guides in a ribonucleoprotein complex were introduced into KOLF2 cells by electroporation/nucleofection as described (Bruntraeger et al., 2019). Cells were plated onto 50 ng/cm^2^ vitronectin (ThermoFisher)‐coated 10 cm dishes in mTesR medium; after 7 days, single colonies were picked and plated across duplicate 96‐well plates, one for genomic DNA extraction using the E.Z.N.A.® Tissue DNA Kit (Omega Bio‐tek), the other for clonal expansion. Clones were screened by PCR of genomic DNA with edit‐flanking primers and separated on 2% agarose gels (unedited clones 733 bp PCR product, edited clones 588 bp product) and confirmed by Sanger sequencing (Eurofins Genomics). Edit‐positive clones were re‐plated onto vitronectin‐coated culture dishes, sub‐colonies picked and expanded.

### 2.9 iPSC differentiation to microglia and culture

Three clones of each iPSC line were selected for differentiation into microglia via the embryoid body (EB) protocol (Figure S5a, Haenseler et al., 2017). Briefly, a single‐cell suspension of iPSC was obtained using TrypLE (ThermoFisher), then seeded at 4 × 10^6^ cells per well in an AggreWell 800 plate (STEMCELL Technologies) in EB medium [mTeSR medium, 50 ng/mL BMP4, 50 ng/mL VEGF, 20 ng/mL SCF (ThermoFisher)] with 10 μM Rho kinase inhibitor (Y‐27632, Abcam, ab120129). The plate was centrifuged at 100 × *g* for 3 min; EBs were grown in the AggreWell plate for 3 days with daily medium changes, then transferred to individual wells of low attachment 6‐well plates (Greiner Bio‐One International). After 3 days of incubation without interference, EBs were harvested and transferred to T‐175 factory flasks (~100 EBS/T175 flask) in factory medium [XVIVO15 (Lonza), GlutaMax 1:100, 100 units/mL penicillin and 100 μg/mL streptomycin (Sigma), 2‐Mercaptoethanol 1:1000, 100 ng/mL M‐CSF, 25 ng/mL IL‐3 (ThermoFisher)]; medium was exchanged weekly. Myeloid precursor cells (MPC), evident after ~2 weeks, were harvested from the culture supernatant and seeded on fibronectin‐coated plates (0.5 μg/cm^2^, STEMCELL Technologies) or poly‐lysine/fibronectin‐coated coverslips (0.1 mg/mL, ThermoFisher; 2 μg/mL, Sigma respectively) for terminal differentiation to microglia in microglial differentiation medium: 1:1 mix of ADF (Advanced DMEM/F12 DMEM (ThermoFisher) supplemented with 2% B27 supplement (ThermoFisher, 17504044), 2 mM GlutaMax, 100 U/mL penicillin, 100 μg/mL streptomycin) and ACM (astrocyte‐conditioned ADF medium; produced in‐house) media. EB factories typically produced viable precursor cells for 4–5 months.

### 2.10 ACM production and characterization

iPSC were differentiated to astrocytes in ADF medium supplemented with ciliary neurotrophic factor as previously described (Maguire et al., 2021). ACM was generated from fully differentiated iPSC‐astrocytes grown in Nunc T‐500 triple layer flasks (Thermo Fisher); ADF medium was removed and replaced with ADF supplemented with 1% vitamin A neurobrew (Miltenyi Biotech). Medium was collected 48 h later, replaced with fresh medium, and stored at −80°C. This cycle was repeated through 6–8 harvests. All harvests were combined to generate a single batch of ACM, which was used for all experiments. Chemokine (C‐C motif) ligand 2 (CCL2; also known as monocyte chemoattractant protein 1) was measured in ACM by ELISA (DuoKit CCL₂, DY279‐05, R&D) and ACM batches were standardized by diluting ACM in ADF to achieve a 1 ng/mL final concentration of CCL2.

### 2.11 qRT‐PCR analysis

qRT‐PCR analysis was performed for assessment of pluripotency and differentiation, SeV clearance, and expression of CR1 and tissue/cell‐specific markers. Total RNA was extracted from cells or tissues using the RNeasy Mini Kit (Qiagen), and 200 ng reverse transcribed with the High‐Capacity RNA‐to‐cDNA™ Kit (ThermoFisher). qRT‐PCR was performed using the QuantStudio™ 7 Flex Real‐Time PCR System (ThermoFisher). All primers were designed to span introns to eliminate amplification of any traces of contaminating DNA (sequences are given in Table S2). Each reaction contained 10 μL PowerUp™ SYBR™ Green Master Mix (ThermoFisher), 1 μL of 10 μM forward primer, 1 μL of 10 μM reverse primer, 1 μL cDNA, and 7 μL H_2_O. The PCR cycle comprised an initial denaturation step of 94°C for 5 min followed by 55 repeated cycles of 94°C for 30 s, 54°C for 45 s, and 72°C for 45 s, with a final cycle of extension at 72°C for 10 min. Melt curve analysis was performed for quality control. Four house‐keeping gene controls, 18 S, SDHA, UBC, and HPRT, were used for quantification. PCR products were confirmed by 2% agarose gel electrophoresis.

### 2.12 Detection of CR1 by immunofluorescence, flow cytometry, and western blotting

Microglia lines, iPSC or iPSC‐microglia grown on sterile glass coverslips were fixed (4% paraformaldehyde), washed in phosphate‐buffered saline (PBS), incubated in blocking buffer [PBS, 3% bovine serum albumin (BSA), 2% normal goat serum; plus 0.1% Triton X‐100 for intracellular staining], then with primary antibody against CR1 or pluripotency/cell markers at optimal dilutions in blocking buffer overnight. Primary monoclonal and polyclonal antibodies (mAb and pAb respectively) against cell‐type markers were anti‐IBA1 (1:100, rabbit mAb, Abcam, ab178846), anti‐GFAP (1:100, rabbit pAb, DAKO, ZO334), anti‐CX3CR1 (1:100, rabbit pAb, Abcam, ab8021), anti‐TMEM119 (1:100, rabbit pAb, Abcam, ab185333), anti‐CD68 (1:100, mouse mAb, Abcam, ab201340), anti‐CD11b (1:100, rat mAb, Abcam, ab8878), anti‐CD45 (1:100, mouse mAb, Abcam, ab30470), anti‐TRA‐1‐60 (1:500, mouse mAb, Abcam, ab16288), anti‐SOX2 (1:1000, mouse mAb, Abcam, ab171380), anti‐SEEA4 (1:500, mouse mAb, Abcam, ab16287), anti‐OCT4 (1:1000, rabbit mAb, Abcam, ab200834), and anti‐HuD + HuC antibody (anti‐HuC/D, rabbit mAb, Abcam, ab184267, EPR19098). Rabbit mAb (Cell Signaling Technology, Inc., 2985 S) and pAb (ThermoFisher, 02–6102) IgG isotype controls were used as negative controls. 6E10 is a recombinant rabbit monoclonal antibody against amyloid β (Enzo Life Sciences, ENZ‐ABS612‐0200). Primary antibodies against CR1 were: affinity‐purified rabbit pAb anti‐CR1 IgG (in house; purified on immobilized sCR1); anti‐CR1 SCR1‐3 mouse mAb 3E10 generated by immunization with recombinant CR1 SCRs 1–3 (Banz et al., 2007); two novel anti‐CR1 mouse mAb, MBI35 and MBI38, generated in house using full‐length sCR1 as immunogen and selected for high affinity binding of CR1. All detected CR1 in immunostaining and western blotting of erythrocytes and for all, specificity was confirmed by demonstrating that pre‐incubation with excess full‐length sCR1 ablated staining (Piddlesden et al., 1994).

Coverslips were washed, incubated with Alexa Fluor conjugated goat anti‐rabbit or anti‐mouse IgG (ThermoFisher) and Hoechst dye, washed, and mounted in ProLong™ Diamond Antifade (ThermoFisher). Fluorescence was imaged by laser scanning microscopy (Leica DMi8) or spinning‐disc microscopy (Zeiss Cell Observer); whole‐cell Z‐stacks were assembled for maximum projections. For double staining, primary antibodies were applied together, detected using appropriately labeled secondary antibodies, and imaged using appropriate filters.

To measure surface markers, cells were incubated on ice with allophycocyanin (APC)‐conjugated anti‐cell antibodies (BioLegend), washed in cold blocking buffer (0.1% BSA in PBS), and analyzed on an Attune NxT flow cytometer (ThermoFisher). Cell populations were selected and analyzed using FlowJo software (BD Biosciences). Negative (no primary) and isotype controls were included.

To detect proteins in situ, brain cryosections (18 μm) from AD cases and controls were rehydrated, fixed in 4% PFA, and permeabilized in PBS with 0.5% Triton‐X100, 5% goat serum, 3% BSA. Primary antibodies were applied overnight, slides washed, and incubated with appropriate secondary antibodies and Hoechst dye in PBS 5% goat serum, 0.5% Triton‐X100. Autofluorescence was blocked with 0.4% Sudan Black B in 70% ethanol prior to washing and mounting. Immunostained tissue sections were digitized using a Zeiss Axioscan z1 Slide Scanner for detection of CR1‐positive cells in cortical gray matter (GM) regions of interest (ROIs). Immunofluorescence quantification and analysis was performed with the researcher blinded to case identity. Image files were processed using QuPath (10.1038/s41598‐017‐17204‐5). The number of anti‐CR1 immunostained cells was measured in ×120 images (field of view [fov] area = 0.3 mm^2^) of ROIs in different brain areas: cortical laminae I‐II (subpial GM); cortical laminae III‐IV (mid GM); and cortical laminae V‐VI (deep GM). CR1‐positive cells were manually tagged in ImageJ (NIH, United States) using the “multipoint” tool and quantified.

For SDS‐PAGE and western blotting, cells were lysed in RIPA buffer (ThermoFisher), protein in the lysate was quantified using the Pierce™ BCA Protein Assay Kit (ThermoFisher) and 30–40 μg was separated on 7% SDS‐PAGE or 3–8% tris‐acetate gels (ThermoFisher) and transferred onto a nitrocellulose membrane. After blocking [3% BSA in PBS 0.1% Tween 20 (PBST)] for 1 h at RT, membranes were incubated with anti‐CR1 primary antibody (2–4 μg/mL) in blocking buffer overnight at 4°C, washed with PBST, then incubated with HRP‐labeled secondary antibody (Jackson Laboratories) in blocking buffer for 1 h at RT. Tubulin staining (1:5000, Abcam, ab7291) was used as an internal standard for protein quantification. Erythrocyte membranes or sCR1 were included for reference. Bands were detected using ECL Detection Reagent (Cytiva), imaged (Syngene, Gbox), and analyzed in ImageJ. To confirm specificity, anti‐CR1 mAbs were pre‐adsorbed with excess sCR1. For gel staining, Coomassie [0.25% (wt/vol) Coomassie Brilliant Blue R‐250, 40% (vol/vol) methanol, 10% (vol/vol) acetic acid] was used.

### 2.13 Bioparticle phagocytosis assay

pHrodo™ Red E. coli BioParticles™ (Invitrogen) were reconstituted in live cell imaging solution (LCI, Invitrogen, P35364) at 1 mg/mL, sonicated and vortexed to disrupt aggregates, added to iPSC‐microglia in 96‐well plates, and phase‐imaged (20×) in an IncuCyte Zoom Live‐Cell Analysis System (Essen BioScience). “Cells only” and “bioparticles only” wells were included. Percentage confluence and total red (internalized bioparticles) object integrated intensity was captured for each well at each time point and the normalized fluorescence reading per well automatically calculated. Cytochalasin D (CytoD; 10 μM) pre‐treated microglia were used as a negative control.

### 2.14 Statistics

All values were expressed as mean ± SEM. Data were plotted using GraphPad Prism v5 and tested for normality using the Shapiro–Wilktest (alpha = .05). Two‐way analysis of variance (ANOVA) with Bonferroni post hoc analysis was used for qRT‐PCR analysis. Nonparametric one‐way ANOVA (Kruskal–Wallis test) using Dunn's multiple comparisons post‐test was used for CR1 brain region analysis (Prism v9; GraphPad, La Jolla, CA). Significance is indicated if *p* < .05.

## 3 RESULTS

### 3.1 CR1 is expressed in HMC3 and IMhu human microglial cell lines

**FIGURE 2:**
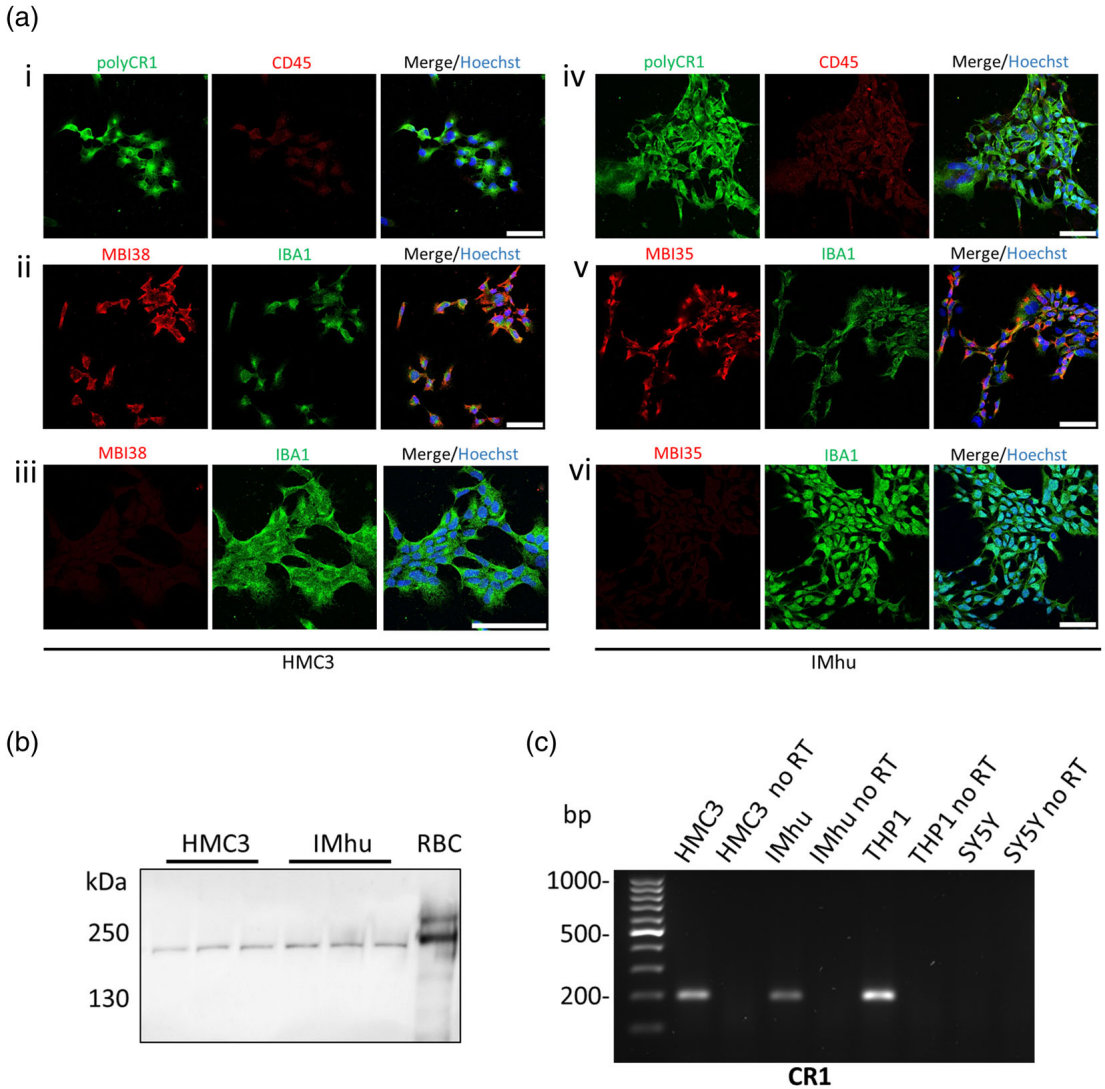
CR1 expression in HMC3 and IMhu microglial cell lines. (a) Dual immunofluorescence staining of HMC3 (i–iii) and IMhu (iv–vi) cells with anti‐CR1 antibodies: affinity‐purified polyclonal anti‐CR1 (i; iv), mAb MBI38 (ii) and mAb MBI35 (v) with microglial markers IBA1 (ii, iii, v, vi) or CD45 (i, iv). Impact of pre‐adsorption with excess sCR1 on staining with mAb MBI38 (iii) and mAb MBI35 (vi) are shown. Nuclei are stained with Hoechst 33342 in the merged images for each set of plates. All images are captured at 40× except set (iii) which are at 80×; scale bar shown in right image for each set is 50 μm in all. (b) CR1 protein was detected in HMC3 and IMhu cells by Western blotting using all anti‐CR1 antibodies (polyclonal shown as example). Protein lysate from red blood cells (RBC) from a CR1*1/CR1*2 donor was used as a positive control. (c) *CR1* mRNA was detected by qRT‐PCR using intron‐spanning primers (Table S2) in HMC3, IMhu, THP‐1 (human monocytic cell line) but not SH‐SY5Y (neuronal) cells. The reaction without reverse transcriptase (no RT) was used as a negative control.

CR1 expression was tested in two widely used human microglial cell lines, HMC3 and IMhu (Figure 2). Immunofluorescence using three well‐characterized anti‐CR1 antibodies demonstrated robust surface expression of CR1 on both lines, co‐localizing with microglial markers IBA1 and CD45 (Figure 2a). Pre‐adsorption of anti‐CR1 antibody with sCR1 abolished staining for each monoclonal antibody on both lines, confirming specificity. CR1 western blotting of cell lysates revealed a single ~190 kDa band in both microglial lines; comparison with bands in CR1*1/CR1*2 erythrocyte lysates showed that both lines were homozygous for CR1*1 (Figure 2b). CR1 mRNA was detected at the expected product size by qRT‐PCR in both HMC3 and IMhu microglial lines and the monocyte cell line, THP1; the neuroblastoma line, SH‐SY5Y, gave no product (Figure 2c).

### 3.2 CR1 is expressed in KOLF2 iPSC‐derived microglia and is not detected in *CR1* knockout cells

**FIGURE 3:**
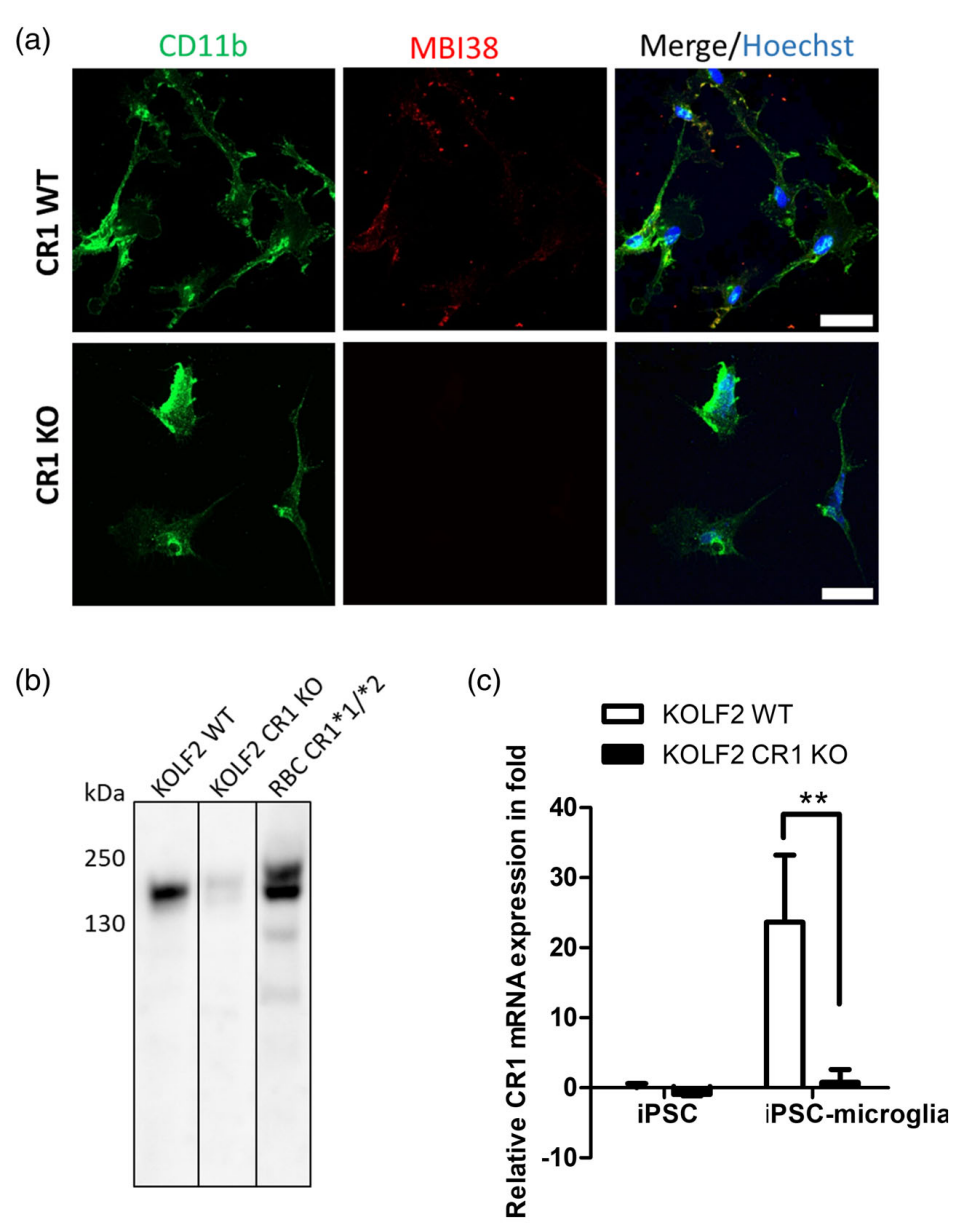
CR1 expression in KOLF2‐derived microglia. (a) CR1 expression in iPSC‐microglia in WT KOLF2‐derived microglia shown by immunofluorescence. CR1 was expressed in a granular, membrane‐associated pattern in KOLF2‐derived microglia but was not detected in *CR1* KO KOLF2. Cells are co‐stained with microglial marker CD11b. Maximum projections of Z‐stacks are presented. Scale bar: 30 μm. (b) CR1 protein was detected in WT iPSC‐microglia by western blotting with the different anti‐CR1 antibodies (polyclonal shown here). Protein lysate from RBC from a CR1*1/CR1*2 donor was used as positive control. CR1 KO cells showed trace staining. (c) *CR1* transcript was quantified by qRT‐PCR using intron‐spanning primers (Table S2) in WT and *CR1* KO KOLF2 iPSC and iPSC‐microglia. Histogram shows the relative mRNA expression normalized to non‐differentiated cells (iPSC); **p < .01.

CR1 expression was assessed in MPC and microglia derived from the human iPSC line, KOLF2, and from KOLF2 in which the CR1 gene had been knocked out (KO) by CRISPR gene editing (CRISPR KO strategy summarized in Figure S1). Three clones for KOLF2 WT and KO lines were differentiated into microglia via EB formation and characterized at the MPC and iPSC‐microglia stages of differentiation for cell‐specific protein markers expression and ability to phagocytose bioparticles (Figure S2). Abundant surface expression of CR1 protein, co‐staining with CD11b, was seen on WT iPSC‐microglia stained with anti‐CR1 while KOLF2 CR1 KO derived microglia, stained with CD11b, were negative, confirming that *CR1* was deleted and further demonstrating specificity of the selected antibodies for the CR1 protein (Figure 3a). Expression of CR1 on KOLF2‐derived microglia and absence in KO lines was also demonstrated by western blotting and qRT‐PCR (Figure 3b,c). Western blot revealed that KOLF2 cells were CR1*1 homozygous. SDS‐PAGE of lysates run in parallel and stained with Coomassie confirmed consistent protein loading.

### 3.3 CR1 expression in donor‐derived MPC and iPSC‐microglia

CR1*1 homozygous (five), heterozygous (seven), and CR1*2 homozygous (four) donors were identified by western blot screening of erythrocyte lysates from 28 donors; data are shown for the first eight donors (Figure S3a). CR1 junction fragment analysis confirmed the western blot results by demonstrating the presence or absence of the CR1*2 allele in these donors (Figure S3b, Kucukkilic et al., 2018). Erythrocyte CR1 density polymorphism was determined by RFLP analysis in 15 donors selected for further study; 10 donors were high‐density allele homozygous (HH) and 5 were heterozygous (HL) (Figure S3c). Due to its status as a strong genetic risk factor for AD (Saunders et al., 1993), donor APOE variant status was tested by RFLP analysis in 27 donors; 22 were ε3/ε3 (Figure S3d). Screening data are summarized in Table S3.

Blood samples were collected from homozygous and heterozygous donors (3 CR1*1/CR1*1; 2 CR1*2/CR1*2; 2 CR1*1/CR1*2), and PBMC were isolated and reprogrammed to iPSC (Figure S4a). Screening data from the donors selected for reprogramming are summarized in Table S4. The iPSC lines were characterized by immunostaining for the pluripotency markers, SSEA4, TRA1‐60 OCT‐4, and SOX‐2 (Figure S4b). Staining of iPSC with anti‐CR1 mAbs was negative, demonstrating that CR1 protein is not expressed in undifferentiated iPSC (Figure S4c). Elimination of SeV was confirmed using qRT‐PCR (Figure S4d).

Three clones for each homozygote and heterozygote line were selected for differentiation into microglia via EB formation (Figure S5a). MPC were large, round cells with filopodia, expressing CD14, CD45, and CD11b (Figure S5b). MPC were further differentiated to iPSC‐derived microglia, confirmed by demonstrating expression of microglia‐specific markers CX3CR1, TMEM119, IBA1, CD45, CD68, and CD11b (Figure S5c). Acquisition of phagocytic capacity was confirmed by demonstrating phagocytosis of pHrodo™ *E. Coli* BioParticles (Figure S5d).

**FIGURE 4:**
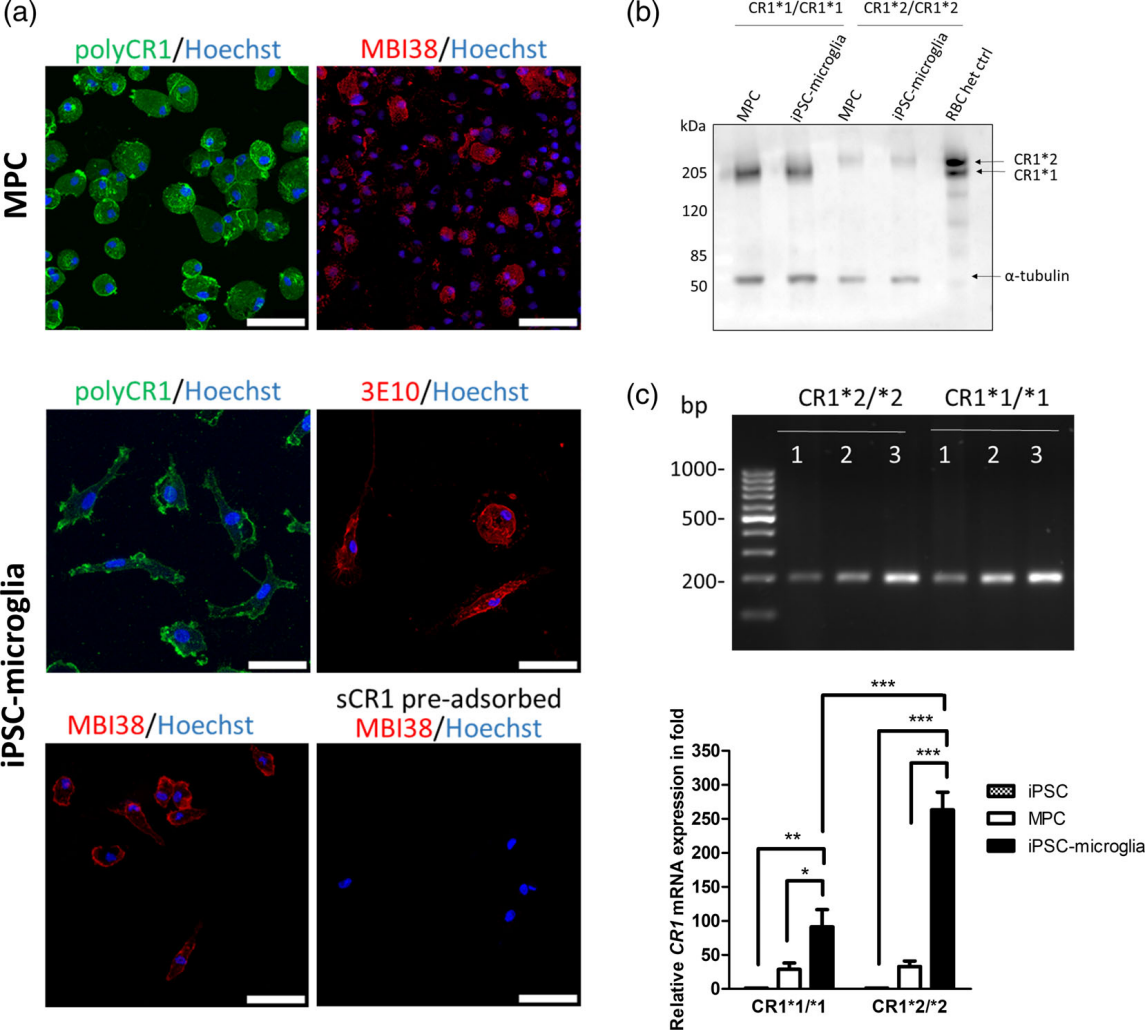
CR1 expression in donor‐derived iPSC‐microglia. (a) CR1 expression in MPC and iPSC‐microglia is shown by immunofluorescence. Cells showed membrane‐associated staining with the pAb against CR1 and the anti‐CR1 mAbs, MBI38, and 3E10; sCR1 pre‐adsorption ablated staining for each antibody (MBI38 shown as example). Maximum projections of Z‐stacks are presented. Representative examples of lines homozygous for CR1*1 and CR1*2 variants are shown. Scale bar: 50 μm. (b) CR1 protein was detected in MPC and iPSC‐microglia expressing the CR1*1 and CR1*2 variants by western blotting with the different anti‐CR1 antibodies (polyclonal anti‐CR1 shown as example). Protein lysate from RBC from a CR1*1/CR1*2 donor was used as a positive control and anti‐tubulin as the loading control. (c) *CR1* transcripts were detected by qRT‐PCR using intron‐crossing primers introns (Table S2) in iPSC (1), MPC (2), and iPSC‐microglia (3) expressing the CR1*1 and CR1*2 variants. Histograms show the relative mRNA expression normalized to non‐differentiated cells (iPSC). *p < .05; **p < .01; ***p < .001.

Surface expression of CR1 was demonstrated on MPC and iPSC‐microglia by staining with anti‐CR1 mAbs and pAb (Figure 4a). CR1 staining localized to the cell membrane, demonstrated by comparing maximum projections of whole cells with mid‐cell Z‐stack images. Staining was abolished when the anti‐CR1 antibody was pre‐adsorbed with sCR1. CR1 expression was confirmed by western blotting of lysates from CR1*1 and CR1*2 lines; sizes of CR1 protein bands were as anticipated in the lines but were consistently weaker in CR1*2 lines (Figure 4b). *CR1* transcripts were detected using qRT‐PCR in CR1*1 and CR1*2 MPC and iPSC‐microglia (Figure 4c). The relative expression of CR1 mRNA was significantly increased in iPSC‐microglia compared to MPC and undifferentiated cells in CR1*1 and CR1*2 expressing cell lines and was significantly higher in CR1*2 compared with CR1*1 lines.

### 3.4 CR1 is expressed on microglia and astrocytes in human brain tissue

**FIGURE 5:**
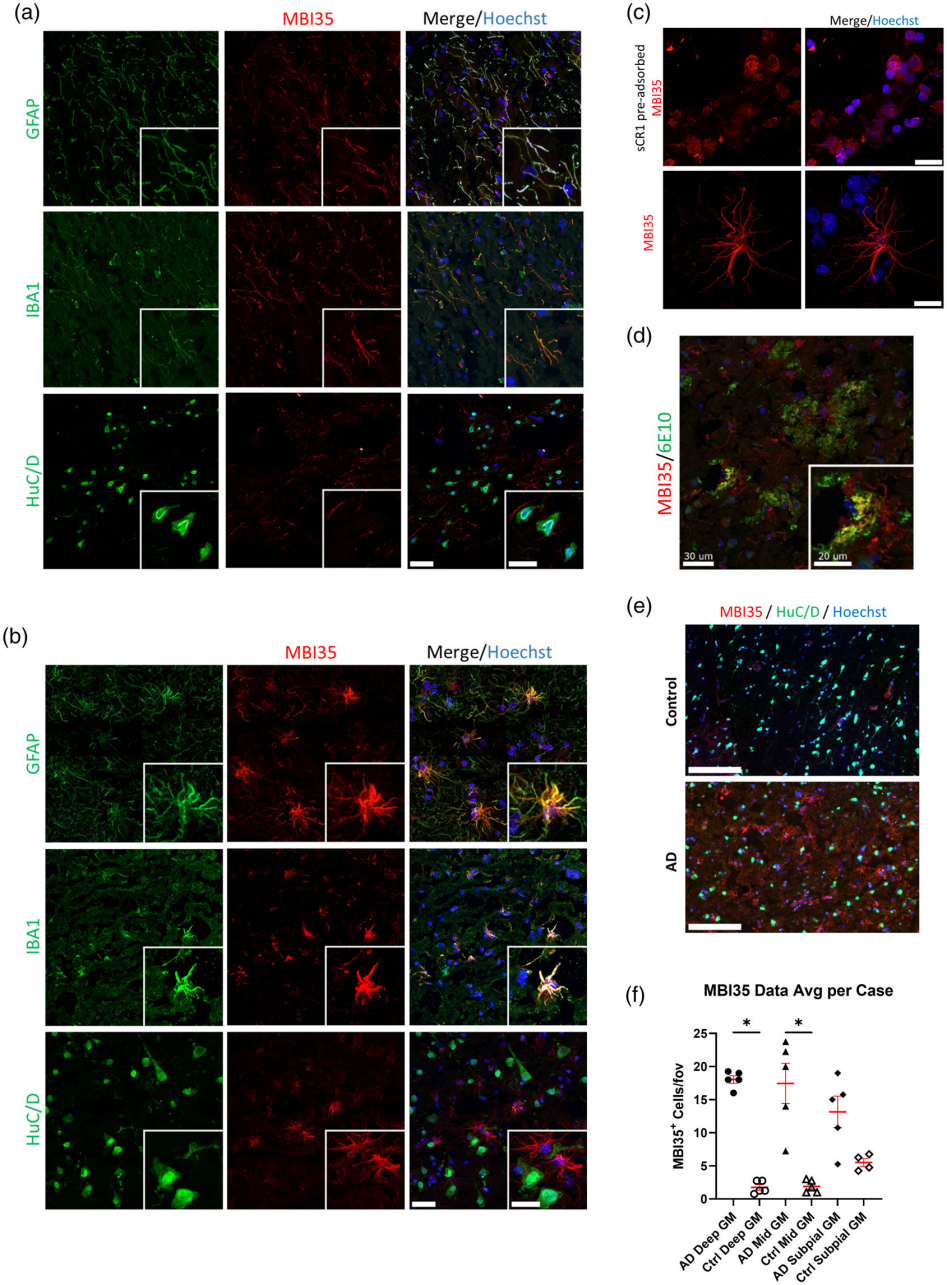
CR1 protein expression in the human brain. Immunofluorescent staining of frozen sections of control (a) and AD (b) human brain tissue (region BA41/42) with anti‐CR1 antibodies (anti‐CR1 mAb MBI35 shown as example) demonstrated expression of CR1 in IBA1‐positive microglia and GFAP‐positive astrocytes but not in HuC/D‐positive neurons. Scale bar: 30 μm in main and 20 μm in insert figures. (c) Pre‐adsorption of MBI35 anti‐CR1 with sCR1 abolished microglial and astroglial staining (scale bars in right panels). Top panel scale bar: 100 μm; bottom scale bar: 30 μm. (d) Immunofluorescent staining of AD human brain tissue with anti‐Aβ (6E10) and anti‐CR1 (MBI35) demonstrated expression of CR1 in Aβ plaques and surrounding glia. Scale bar: 30 μm in main and 20 μm in insert figure. (e) Representative low power views (scale bar: 150 μm) of whole sections to show CR1 staining in AD and control brain gray matter (GM). Staining was clearly increased in AD cases compared to controls. (f) Quantitation of MBI35‐positive cell density per field of view (fov) in GM showed increased staining in AD brain in all ROIs, significantly greater compared to controls in deep and mid GM ROIs. *p < .05; data from five AD and five control brains.

**FIGURE 6:**
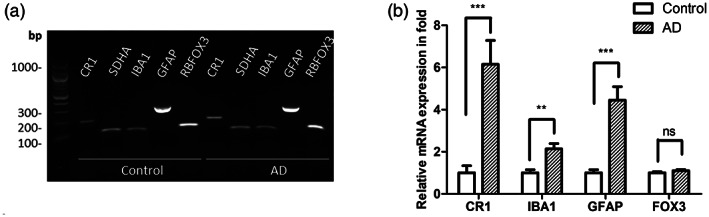
CR1 mRNA expression in the human brain. (a) Agarose gel demonstrating detection of *CR1* transcripts in control and AD human brain tissue using specific primers. Transcripts for the cell type‐specific markers *IBA1*, *GFAP*, and *RBFOX3* and the housekeeping gene SDHA were also present in the brain extracts. (b) Relative *CR1*, *IBA1*, *GFAP* and *RBFOX3* mRNA expression (‐fold) in control and AD human brain tissue; CR1 mRNA showing ~5‐fold greater expression in AD brain. **p < .01; ***p < .001, data from five AD and five control brains.

Frozen post‐mortem brain samples (region BA41/42) obtained from five AD cases (Braak VI) and five age and sex‐matched controls (Table S1) were tested for CR1 expression by immunohistochemistry. Representative examples of staining obtained using the anti‐CR1 mAb MBI35 are shown in Figure 5a,b. In all cases (Figure 5b) and controls (Figure 5a) and with all tested anti‐CR1 antibodies, cell‐specific staining for CR1 was seen. Double staining showed that both IBA1‐positive microglia (stained with rabbit mAb ab178846) and GFAP‐positive astrocytes (stained with rabbit pAb ZO334) expressed CR1 protein (mouse mAb MBI35), while HuC/D‐positive neurons (stained with rabbit mAb ab184267) were negative. Antibody pre‐adsorption with sCR1 abolished specific staining (Figure 5c) and isotype controls for the rab mAb and pAb were negative (Figure S6). Staining with anti‐Aβ (6E10) and anti‐CR1 (MBI35) demonstrated expression of CR1 in Aβ plaques and surrounding glia (Figure 5d). CR1‐positive cells were quantified in deep, mid, and subpial cortical GM in all cases and controls. Neuronal HuC/D was used to identify and enumerate CR1‐negative neurons. CR1‐positive cell number was markedly higher in AD cases compared to controls in all areas, significantly in deep and mid GM ROIs (Table S1, Figure 5e,f). qRT‐PCR analysis was performed on tissue‐extracted RNA for assessing expression of CR1 and tissue/cell‐specific markers. CR1 transcripts were detected in all control and AD whole brain extracts tested (Figure 6a). Transcripts for microglial (IBA1), astrocyte (GFAP) and neuronal (RBFOX3) and an endogenously expressed control (SDHA) were also detected (Figure 6a). CR1 mRNA expression was significantly increased (~5‐fold) in the AD samples compared to controls (Figure 6b). Expression of mRNA for the microglial marker IBA1 and the astrocytic marker GFAP were also significantly increased in AD compared to control samples, indicative of pathology‐associated microgliosis and astrogliosis; in contrast, expression of mRNA for the neuronal marker, RBFOX3, was similar in control and AD samples (Figure 6c).

## 4 DISCUSSION

Over the last 20 years, an abundance of evidence has implicated complement in AD pathogenesis (reviewed in Morgan, 2018). GWAS have identified AD risk single nucleotide polymorphisms in complement genes (reviewed in Torvell et al., 2021), including *CR1*, *CLU*, and more recently a suggestive association in *C1S* (Bellenguez et al., 2022). Biomarker studies have also identified alterations in complement proteins and activation products in blood and/or cerebrospinal fluid that distinguish controls from mild cognitive impairment and are predictive of progression to AD (Hakobyan et al., 2016; Morgan et al., 2019). In post‐mortem immunohistochemical studies of AD brain, C1q, C4b, C3b/iC3b, and membrane attack complex (MAC) have all been shown to co‐localize with plaques and tangles (Ishii & Haga, 1984; Rogers et al., 1992; Veerhuis et al., 2003). Data from animal models have implicated complement in amyloid clearance and in synapse loss (Maier et al., 2008; Shi et al., 2017; Wyss‐Coray et al., 2002); notably, our recent study demonstrated that MAC formation is an important driver of synapse loss in AD models (Carpanini et al., 2022).

Although long established among the top AD‐associated GWAS hits, the specific cellular and molecular roles of CR1 in brain health and disease are poorly understood. Improved understanding of the expression and roles of CR1 in the brain in health and disease is needed to explain its association with AD and to facilitate rational design of diagnostic or therapeutic tools. Complement inhibition is a proven treatment in numerous diseases, including the neurological diseases, neuromyelitis optica and myasthenia gravis (Huda et al., 2014; Pittock et al., 2019). In neurodegeneration, anti‐complement drugs are in clinical trials for treatment of Huntington's disease (NCT04514367) and amyotrophic lateral sclerosis (NCT04248465). Implicating *CR1* expression and complement dysregulation as players in AD pathogenesis could signpost new treatment strategies.

Expression of CR1 in the brain has been the subject of contrasting data and debate and published reports of CR1 protein and message in the brain and on brain cells are confusing and contradictory. In early studies, protein expression was variously reported as only in astrocytes (Gasque et al., 1996), restricted to phagocytic Kolmer cells of the choroid plexus and ependymal cells (Canova et al., 2006), no expression at all (Singhrao et al., 1999), or on choroid plexus, microglia and neurons in AD and control brain tissue (Hazrati et al., 2012). A comprehensive analysis of CR1 staining tested a panel of seven mAbs and two antisera from various sources in formalin‐fixed AD and control brain (Fonseca et al., 2016); while most of these reagents did not stain the tissue, two mAbs (8C9.1 and J3B11) from the panel specifically stained astrocytes and specificity was confirmed by pre‐adsorption with sCR1 and staining of isolated astrocytes. In contrast, a subsequent report from the same investigators tested four mAbs against CR1, not including the two shown in their earlier publication, and found no specific staining in brain parenchyma although vasculature was stained (Johansson et al., 2018). *CR1* mRNA expression in AD and control brain has been widely reported, including the demonstration that CR1 expression levels were increased in AD brain and associated with cognitive score (Karch et al., 2012) and that CR1 gene expression levels strongly associated with AD risk (Allen et al., 2015). Microarray data from the Allen Human Brain Atlas also demonstrates significant CR1 expression in brain (Hawrylycz et al., 2012). A recent report described *CR1* mRNA expression in fetal and iPSC‐derived microglia (Haenseler et al., 2017).

Although the published evidence (with a few notable exceptions) supports CR1 gene expression in brain, CR1 protein expression remains an area of controversy, one that requires clarification if we are to understand how CR1, a major GWAS hit in AD, influences disease risk. The aim of the current study was to resolve the CR1 expression controversy by adopting a multi‐pronged approach to explore CR1 expression in established microglial cell lines, iPSC‐derived and primary microglia, and human brain tissue. First, we demonstrated CR1 expression in two well‐characterized human microglial cell lines, HMC3 and IMhu cell lines. *CR1* transcripts and protein were detected by qRT‐PCR and immunofluorescence respectively. Protein was also detected by western blotting, revealing that both lines were homozygous for the common CR1*1 variant. Next, we tested the expression of CR1 in iPSC‐derived microglia. We used two iPSC sources, the iPSC reference line, KOLF2, and iPSC lines generated from donors carrying known CR1*1 and CR1*2 variant alleles. Undifferentiated KOLF2 cells expressed trace amounts of CR1 mRNA and no detectable protein, while KOLF2‐derived MPC and microglia both expressed the CR1*1 variant mRNA and protein, microglia to a much greater degree. Similarly, donor‐derived iPSC lines were CR1 protein negative and expressed trace amounts of mRNA prior to differentiation, while MPC and, to a greater extent, microglia, strongly expressed CR1 mRNA and protein. *CR1* gene expression was upregulated >90‐fold in fully differentiated iPSC‐microglia compared to MPC. In both KOLF2 and donor‐derived iPSC microglia, imaging showed abundant cell surface CR1 expression. All tested anti‐CR1 antibodies gave similar staining patterns; specificity of staining was demonstrated by pre‐adsorption of antibody with sCR1, and for KOLF2‐derived microglia, by CRISPR deletion of *CR1*. Finally, we used the same panel of anti‐CR1 antibodies and controls to demonstrate expression of CR1 in situ in the human brain. Specific staining was seen in control and AD brain sections; numbers of CR1‐positive cells were ~5‐fold higher in AD GM regions compared to non‐AD controls. Double‐staining with cell‐type specific markers confirmed that not only microglia but also astrocytes were CR1‐positive, the latter particularly in the AD brain. Of note, neurons (stained with HuC/D) were negative in all samples. *CR1* transcript was also detected in both control and AD human brain tissue extracts and expression was ~6‐fold higher in AD tissue compared to control.

We speculate that failure to detect CR1 protein expression in some studies may be the result of tissue selection and processing (we used frozen tissue and optimized post‐fixation conditions), choice of antibodies (we used well‐characterized mAbs raised against full‐length sCR1 pre‐selected for high binding affinity and an affinity‐purified polyclonal) and lack of relevant controls. The same factors are likely to be responsible for the different cell specificities in those studies that do report CR1 protein expression in brain.

Taken together, our multisource data incontrovertibly demonstrate expression of CR1 message and protein in glial cells and in the brain. Our initial focus was on microglia, but the demonstration that astrocytes also express CR1 opens new avenues of work, particularly relevant given the critical roles of astrocytes in synaptic elimination (Lee et al., 2021). Our ambition is to explain how the CR1*2 variant confers AD risk, and to this end, we have generated CR1*1/CR1*1, CR1*1/CR1*2, and CR1*2/CR1*2 iPSC lines. These iPSC lines provide us with essential tools to study the functional differences between risk and non‐risk variants that cause the association with AD risk. Here we show that all variant combinations were expressed and membrane‐localized on iPSC microglia. Our initial characterization suggests reduced CR1 protein expression in CR1*2/CR1*2 compared to CR1*1/CR1*1 iPSC microglial lines despite increased *CR1* mRNA in the former. This interesting disconnect is the subject of ongoing work that will extend the expression analysis to define precisely how the variants affect CR1 levels and distribution on iPSC‐derived microglia and astrocytes and explore the impact of the *CR1* variants on C3 fragment processing and phagocytic capacity for relevant targets. Detailed understanding of the impact of *CR1* variants on interactions with its partners in the complement system and elsewhere will inform disease mechanism and signpost routes to novel disease modifying therapies.

## 5 CONCLUSIONS

CR1 transcript and protein are expressed in human microglia ex vivo and on microglia and astrocytes in situ in the human brain. The findings support the hypothesis that *CR1* variants affect AD risk by directly impacting glial functions.

## AUTHOR CONTRIBUTIONS

Nikoleta Daskoulidou led the experimental work. Bethany Shaw conducted the KOLF2 component of the work. B. Paul Morgan and Nicholas D. Allen designed and supervised the project. All other authors Megan Torvell, Lewis Watkins, Emma L. Cope, and Sarah M. Carpanini contributed to aspects of the experimental work. Nikoleta Daskoulidou drafted the manuscript. B. Paul Morgan finalized the submission. All authors read and approved the final manuscript.

## FUNDING INFORMATION

This work is supported by the UK Dementia Research Institute which receives its funding from UK DRI Ltd, funded by the UK Medical Research Council, Alzheimer's Society and Alzheimer's Research UK, and by Alzheimer's Society Project Grant (AS‐PG‐17‐005).

## CONFLICT OF INTEREST STATEMENT

B. Paul Morgan serves as a consultant for Kira Pharmaceuticals and as an advisory board member for Complement Therapeutics.

## CONSENT FOR PUBLICATION

All authors have approved this manuscript and consented to its submission for publication.

## Supporting information


**FIGURE S1. Generation of CR1 KO KOLF2 CRISPR clones**. (a) *CR1* CRISPR KO design is illustrated using SnapGene. Two guide RNAs within *CR1* exon 2 were selected to introduce a large deletion (shown by the red bar) and resulting in a frame shift and introduction of a premature stop codon. (b) Initial PCR screening using primers flanking the area of the deletion. Non‐edited clones give a band at 733 bp while edited clones give a band at 588 bp. Clone D2 was not edited; clones D8 and G3 were homozygous *CR1* KO; clones D3, D7, D9, E2, F2, G2, and G4 were heterozygous KOs. (c) Subclones from homozygous *CR1* KO clones were picked and PCR screened to confirm KO. (d) *CR1* homozygous KO sequenced across the deletion site. The table summarizes the nucleotide and amino acid sequences resulting from these edits. Compared to the unedited KOLF2 sequence, *CR1* KO clone C3 showed two different cut sequences in the two alleles, while D8 and G3 showed the same cut on both alleles. The amino acid sequence produced from the edited gene is shown; ‘*’ indicates a STOP codon in all three clones, in exon 2 for C3 and exon 3 for D8 and G3.

**FIGURE S2. Differentiation of KOLF2 iPSC to microglia**. (a) Staining of KOLF2‐derived MPC for the myeloid lineage markers CD11b and CD45. Scale bar: 30 μm. (b) Staining of WT KOLF2‐derived microglia for the microglial markers CD68 and CX3CR1. Scale bar: 30 μm. (c) Staining of *CR1* KO KOLF2‐derived microglia for the microglial markers CD68 and CX3CR1 (clone G3 shown). Scale bar: 30 μm. (d) KOLF2‐derived iPSC‐microglia are phagocytosis‐competent as assessed by uptake of pHrodo *E. coli* bioparticles. Representative pictures of the cells at 0 and 6 h incubation. Cells treated with cytochalasin D (CytoD) 10 μM were used as a negative control.

**FIGURE S3. Screening of healthy donors for *CR1* isoforms and *APOE* status**. (a) Representative results of a western blot screen for CR1 protein isoforms expressed in erythrocyte membranes prepared from blood donated from eight healthy donors. Three in‐house antibodies were used: mouse anti‐human CR1 mAbs MBI38 and MBI35 and a rabbit anti‐human polyclonal against CR1. (b) Confirmation of expression of CR1*2 in the above samples using CR1 junction PCR. A PCR product is generated when the LHR‐S domain is present as shown in the schematic. (c) Representative results of screening for the density polymorphism of CR1 using HindIII‐specific PCR. A represents HindIII‐digested and B non‐HindIII‐digested PCR products. H/H, H/L and L/L were control samples of known status. Different genotypes yield different band patterns (1, 2, and 3 in right hand panel) on digestion. (d) Representative results for donor screening for *APOE* status using HhaI‐specific PCR on a 4% agarose gel. Genotyping results are shown in the table, typed according to Ingelsson et al. (2003).

**FIGURE S4. Donor PBMC reprogramming to iPSC and characterization**. (a) Procedure (modified from www.thermofisher.com) and representative pictures of the reprogramming of PBMC to iPSC using the CytoTune™‐iPS 2.0 Sendai Reprogramming Kit. (b‐c) iPSC are positive for the pluripotency markers, transcription factors Oct‐4 and SOX2, and plasma membrane receptors, TRA1‐60 and SEEA4, and negative for CR1 expression as tested using three anti‐CR1 antibodies, MBI38, MBI35, and polyclonal. Scale bar: 50 μm. (d) Clearance of SeV was confirmed by qRT‐PCR. Cells were tested at passage numbers 3 to 7 and compared to post‐transduction cells (positive control 1 and 2). RNA from HMC3 cells was used as a negative control.

**FIGURE S5. Differentiation of donor‐derived iPSC to microglia and characterization**. (a) Procedure of the differentiation of iPSC to iPSC‐microglia using the embryoid body protocol (Haenseler et al., 2017). (b) Characterization of MPC: (i) flow cytometry showing cell surface expression of CD45, CD14, and CD11b in microglial precursors; (ii) immunofluorescence showing expression of CD11b and CD45 in microglial precursors. Scale bar: 50 μm. (c) Characterization of KOLF2 iPSC‐microglia by immunofluorescence for the expression of macrophage and microglial‐specific markers including TMEM119, CX3CR1, IBA1, CD45, CD68 and CD11b. Scale bar: 50 μm. (d) Functional characterization of iPSC‐microglia by demonstration of phagocytosis‐competence using pHrodo *E. coli* bioparticles. Representative pictures of the cells at 0 and 40 min incubation time. Cells pre‐treated with CytoD 5 μM were used as a negative control.

**FIGURE S6. Staining of human AD brain tissue with antibodies against cell‐specific proteins and isotype controls**. Staining with anti‐IBA1 (rabbit mAb ab178846) for microglia, anti‐GFAP (rabbit pAb ZO334) for astrocytes and anti‐HuC/D (rabbit mAb ab184267) for neurons. Staining with isotype controls (IC) for the rab mAb and pAb and with anti‐rabbit secondary were negative.

**TABLE S1.** Human brain tissue information
*Note: Comparison between respective deep*, *mid*, and subpial GM regions of the control and AD human brain tissue. * P < 0.05
**TABLE S2.** qRT‐PCR primer list
**TABLE S3.** Results from screening of donors for CR1 length polymorphism, CR1 density polymorphism, and APOE status. 28 donors were screened for the density polymorphism of CR1, 15 of them for the CR1 density polymorphism and 27 for their APOE status. Representative examples of 8 donors screening are presented in Figure S3.
**TABLE S4.** List of the generated iPSC lines (+KOLF2) and their CR1 length and density polymorphism genotypes and APOE status.

## Data Availability

Primary data and materials described are available on reasonable request for academic use.
