# Supplementary figures and images for: Complement receptor 1 is expressed on brain cells and in the human brain
Source: Glia. 2023 Feb 24;71(6):1522–35. doi: 10.1002/glia.24355 (PMC10953339; doi:10.1002/glia.24355)

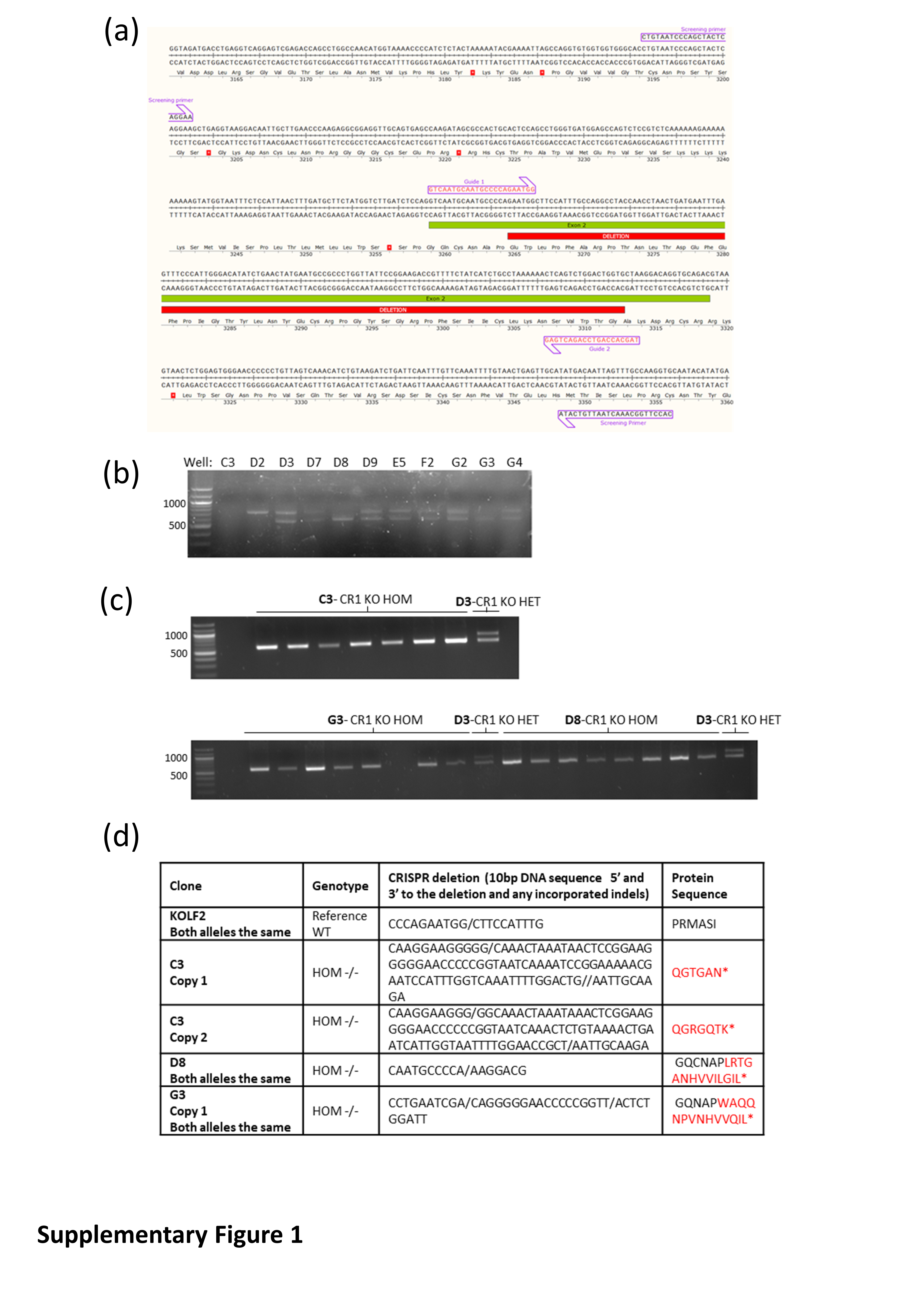

Supplement: Supplementary file 1 — FIGURE S1. Generation of CR1 KO KOLF2 CRISPR clones. (a) CR1 CRISPR KO design is illustrated using SnapGene. Two guide RNAs within CR1 exon 2 were selected to introduce a large deletion (shown by the red bar) and resulting in a frame shift and introduction of a premature stop codon. (b) Initial PCR screening using primers flanking the area of the deletion. Non‐edited clones give a band at 733 bp while edited clones give a band at 588 bp. Clone D2 was not edited; clones D8 and G3 were homozygous CR1 KO; clones D3, D7, D9, E2, F2, G2, and G4 were heterozygous KOs. (c) Subclones from homozygous CR1 KO clones were picked and PCR screened to confirm KO. (d) CR1 homozygous KO sequenced across the deletion site. The table summarizes the nucleotide and amino acid sequences resulting from these edits. Compared to the unedited KOLF2 sequence, CR1 KO clone C3 showed two different cut sequences in the two alleles, while D8 and G3 showed the same cut on both alleles. The amino acid sequence produced from the edited gene is shown; ‘*’ indicates a STOP codon in all three clones, in exon 2 for C3 and exon 3 for D8 and G3. [file GLIA-71-1522-s001.tif]

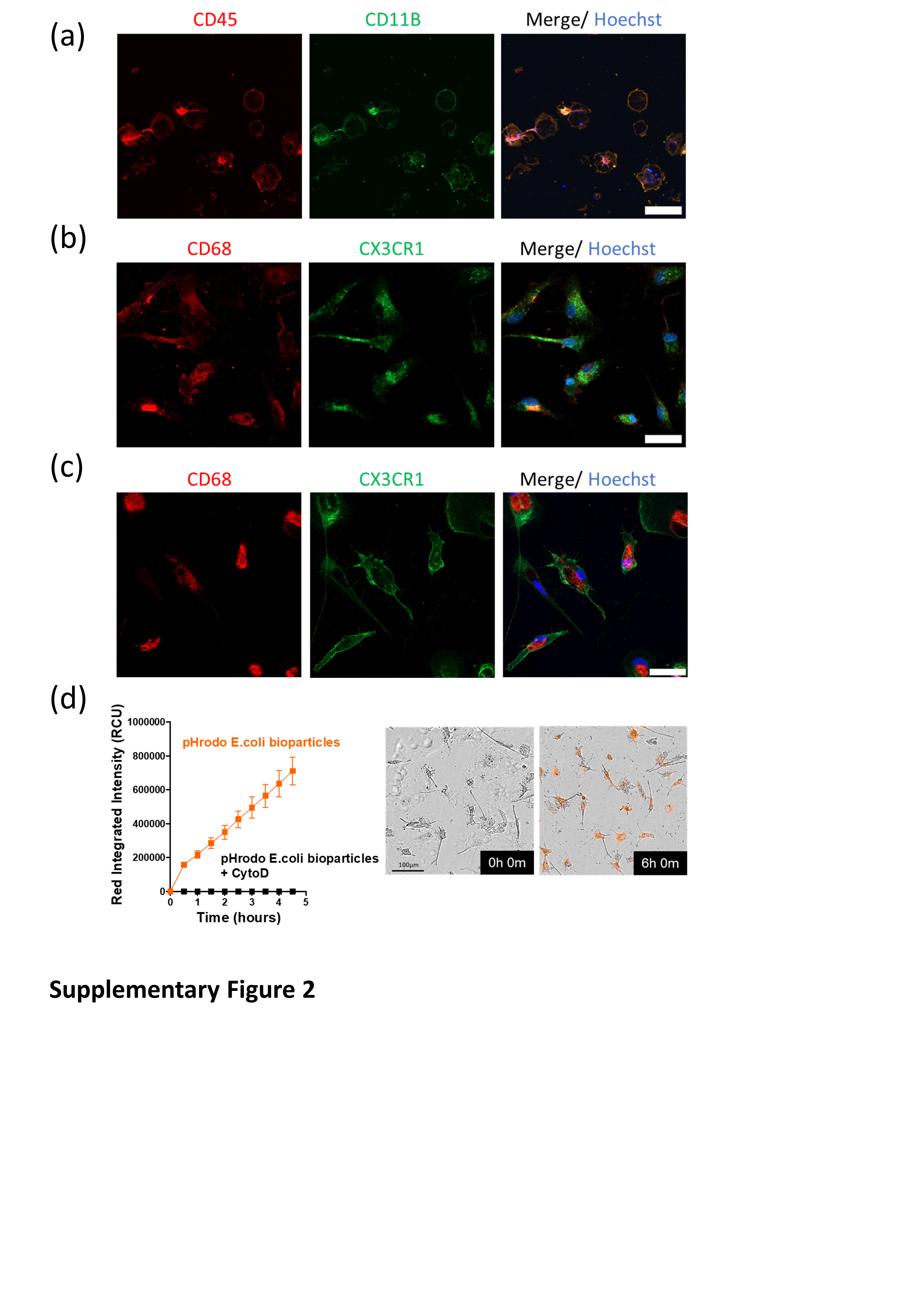

Supplement: Supplementary file 2 — FIGURE S2. Differentiation of KOLF2 iPSC to microglia. (a) Staining of KOLF2‐derived MPC for the myeloid lineage markers CD11b and CD45. Scale bar: 30 μm. (b) Staining of WT KOLF2‐derived microglia for the microglial markers CD68 and CX3CR1. Scale bar: 30 μm. (c) Staining of CR1 KO KOLF2‐derived microglia for the microglial markers CD68 and CX3CR1 (clone G3 shown). Scale bar: 30 μm. (d) KOLF2‐derived iPSC‐microglia are phagocytosis‐competent as assessed by uptake of pHrodo E. coli bioparticles. Representative pictures of the cells at 0 and 6 h incubation. Cells treated with cytochalasin D (CytoD) 10 μM were used as a negative control. [file GLIA-71-1522-s002.tif]

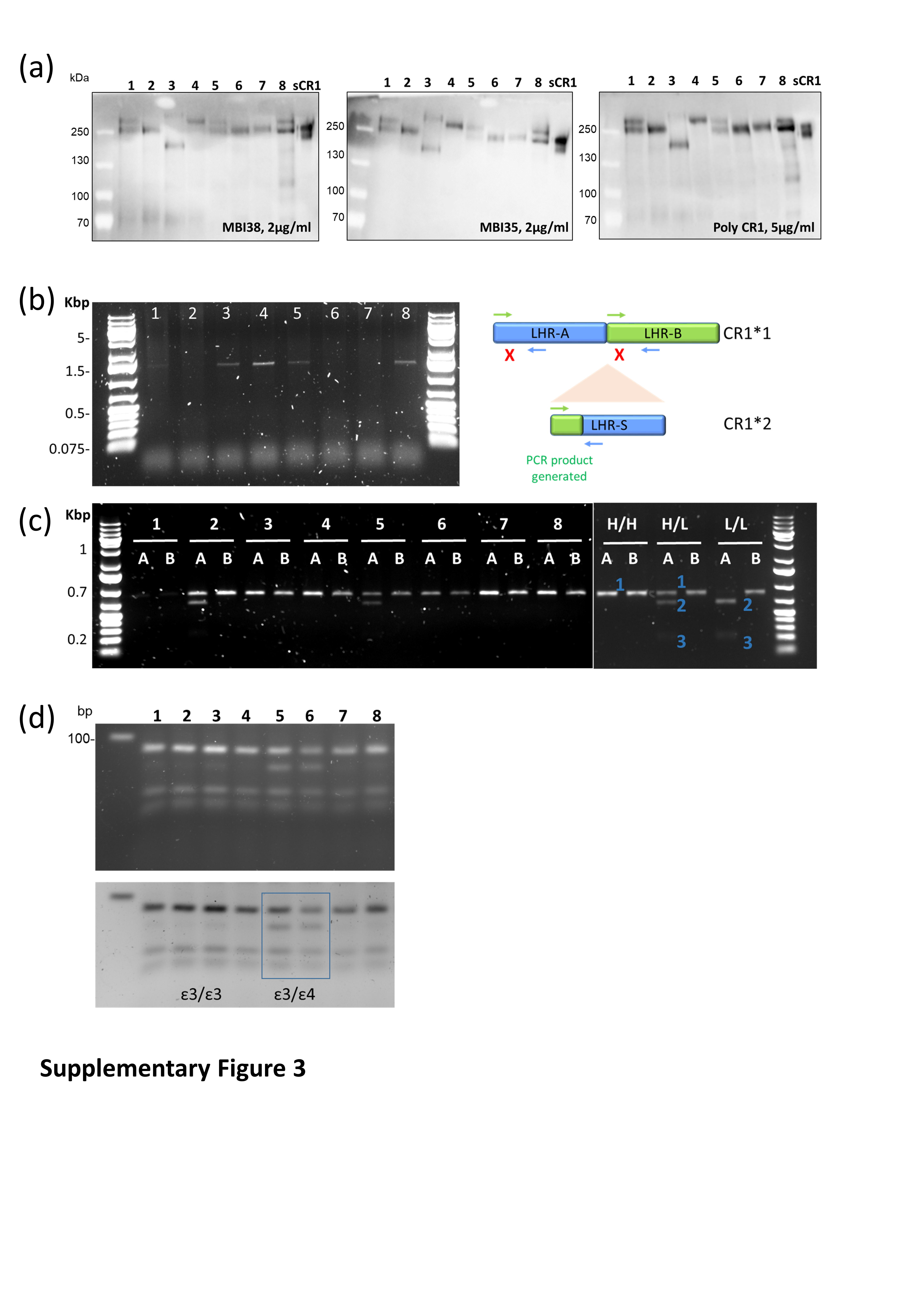

Supplement: Supplementary file 3 — FIGURE S3. Screening of healthy donors for CR1 isoforms and APOE status. (a) Representative results of a western blot screen for CR1 protein isoforms expressed in erythrocyte membranes prepared from blood donated from eight healthy donors. Three in‐house antibodies were used: mouse anti‐human CR1 mAbs MBI38 and MBI35 and a rabbit anti‐human polyclonal against CR1. (b) Confirmation of expression of CR1*2 in the above samples using CR1 junction PCR. A PCR product is generated when the LHR‐S domain is present as shown in the schematic. (c) Representative results of screening for the density polymorphism of CR1 using HindIII‐specific PCR. A represents HindIII‐digested and B non‐HindIII‐digested PCR products. H/H, H/L and L/L were control samples of known status. Different genotypes yield different band patterns (1, 2, and 3 in right hand panel) on digestion. (d) Representative results for donor screening for APOE status using HhaI‐specific PCR on a 4% agarose gel. Genotyping results are shown in the table, typed according to Ingelsson et al. (2003). [file GLIA-71-1522-s006.tif]

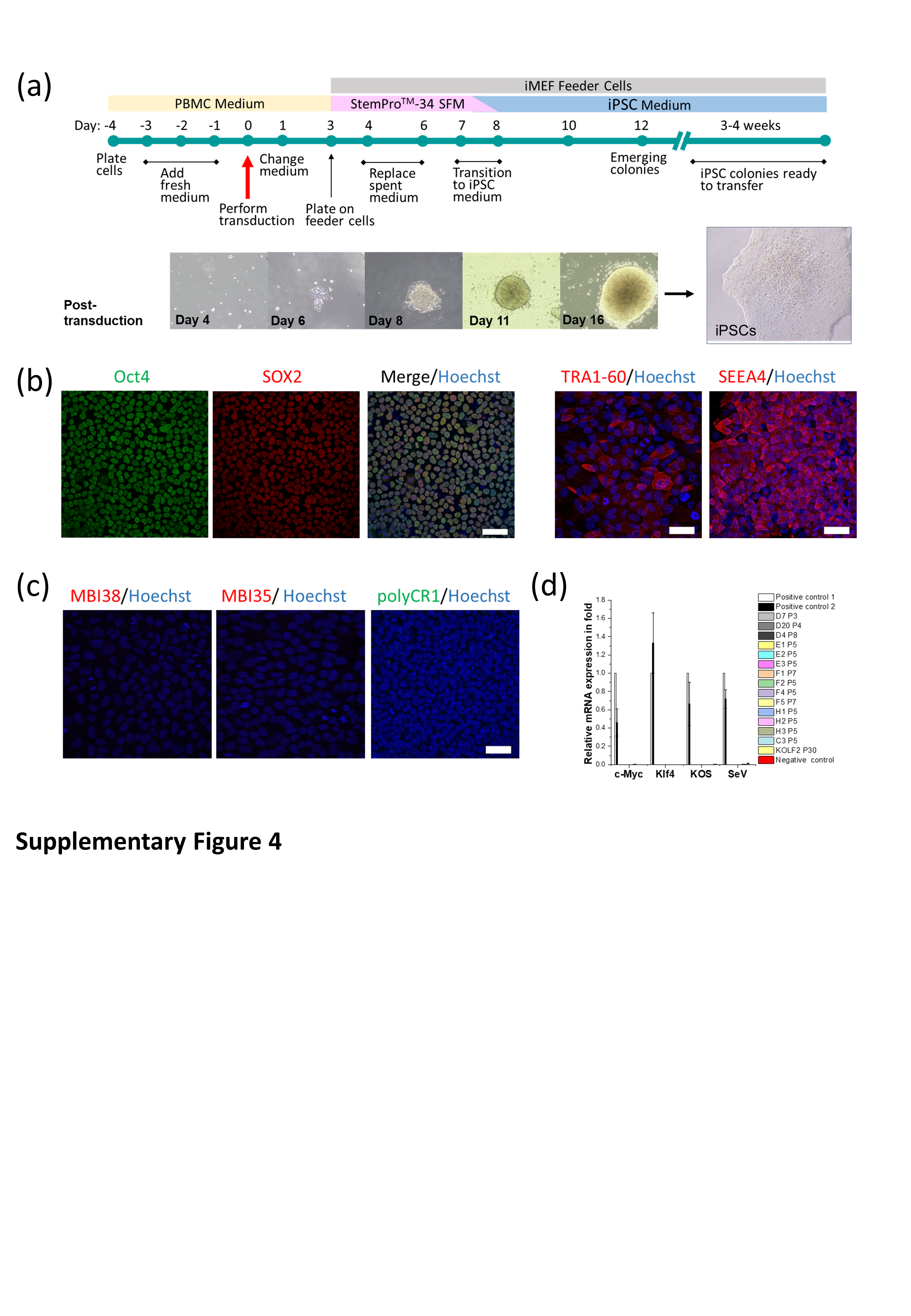

Supplement: Supplementary file 4 — FIGURE S4. Donor PBMC reprogramming to iPSC and characterization. (a) Procedure (modified from www.thermofisher.com) and representative pictures of the reprogramming of PBMC to iPSC using the CytoTune™‐iPS 2.0 Sendai Reprogramming Kit. (b‐c) iPSC are positive for the pluripotency markers, transcription factors Oct‐4 and SOX2, and plasma membrane receptors, TRA1‐60 and SEEA4, and negative for CR1 expression as tested using three anti‐CR1 antibodies, MBI38, MBI35, and polyclonal. Scale bar: 50 μm. (d) Clearance of SeV was confirmed by qRT‐PCR. Cells were tested at passage numbers 3 to 7 and compared to post‐transduction cells (positive control 1 and 2). RNA from HMC3 cells was used as a negative control. [file GLIA-71-1522-s005.tif]

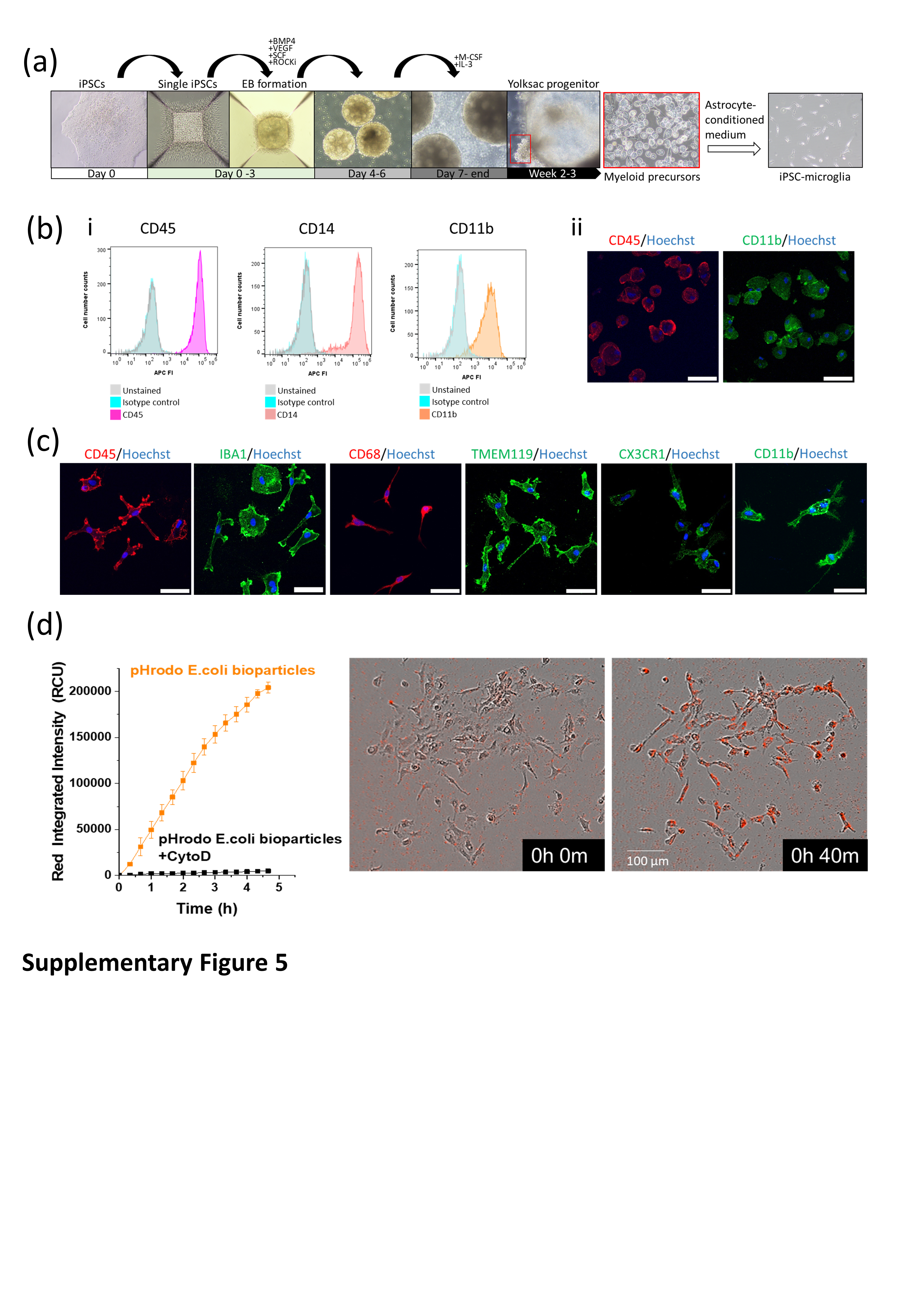

Supplement: Supplementary file 5 — FIGURE S5. Differentiation of donor‐derived iPSC to microglia and characterization. (a) Procedure of the differentiation of iPSC to iPSC‐microglia using the embryoid body protocol (Haenseler et al., 2017). (b) Characterization of MPC: (i) flow cytometry showing cell surface expression of CD45, CD14, and CD11b in microglial precursors; (ii) immunofluorescence showing expression of CD11b and CD45 in microglial precursors. Scale bar: 50 μm. (c) Characterization of KOLF2 iPSC‐microglia by immunofluorescence for the expression of macrophage and microglial‐specific markers including TMEM119, CX3CR1, IBA1, CD45, CD68 and CD11b. Scale bar: 50 μm. (d) Functional characterization of iPSC‐microglia by demonstration of phagocytosis‐competence using pHrodo E. coli bioparticles. Representative pictures of the cells at 0 and 40 min incubation time. Cells pre‐treated with CytoD 5 μM were used as a negative control. [file GLIA-71-1522-s007.tif]

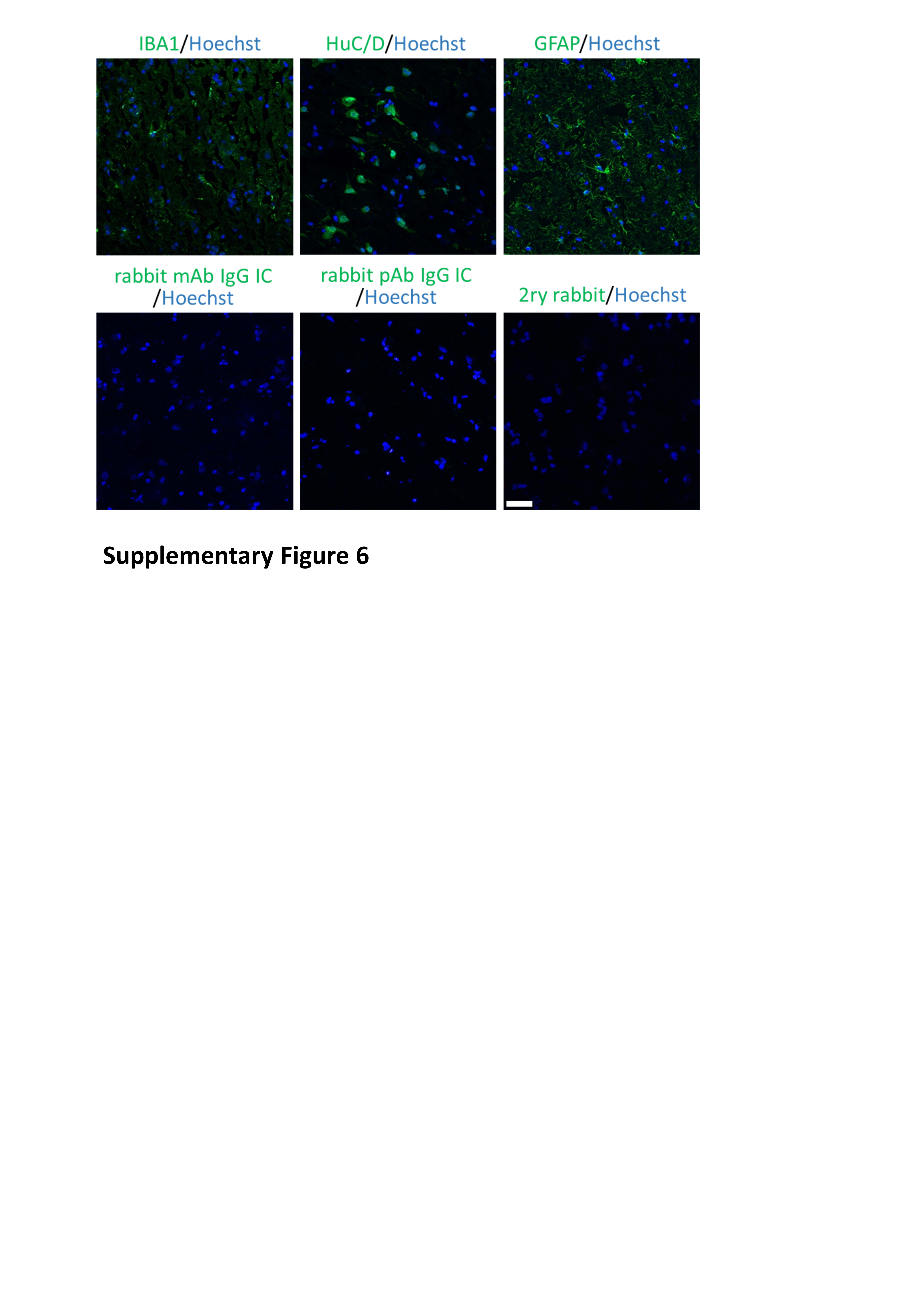

Supplement: Supplementary file 6 — FIGURE S6. Staining of human AD brain tissue with antibodies against cell‐specific proteins and isotype controls. Staining with anti‐IBA1 (rabbit mAb ab178846) for microglia, anti‐GFAP (rabbit pAb ZO334) for astrocytes and anti‐HuC/D (rabbit mAb ab184267) for neurons. Staining with isotype controls (IC) for the rab mAb and pAb and with anti‐rabbit secondary were negative. [file GLIA-71-1522-s003.tif]
