# Supplementary material for: Complement receptor 1 is expressed on brain cells and in the human brain
Source: Glia. 2023 Feb 24;71(6):1522–35. doi: 10.1002/glia.24355 (PMC10953339; doi:10.1002/glia.24355)
Supplement: Supplementary file 7 — TABLE S1. Human brain tissue information Note: Comparison between respective deep, mid, and subpial GM regions of the control and AD human brain tissue. * P < 0.05 TABLE S2. qRT‐PCR primer list TABLE S3. Results from screening of donors for CR1 length polymorphism, CR1 density polymorphism, and APOE status. 28 donors were screened for the density polymorphism of CR1, 15 of them for the CR1 density polymorphism and 27 for their APOE status. Representative examples of 8 donors screening are presented in Figure S3. TABLE S4. List of the generated iPSC lines (+KOLF2) and their CR1 length and density polymorphism genotypes and APOE status. [file GLIA-71-1522-s004.docx]

Supplementary Table 1 Human brain tissue information

| **ID** | **BNN** | **AD/**  **control** | **Sex** | **Age (years)** | **Post-mortem**  **Delay (hours):** | **MBI35**  **quantification**  (avg MBI35^+^ cells/fov) | Deep  GM | Mid  GM | Subpial  GM |
| --- | --- | --- | --- | --- | --- | --- | --- | --- | --- |
| SD032/16 | BBN001.29084 | Control | M | 58 | 104 |  | 1.25 | 1.75 | 6.75 |
| SD035/15 | BBN001.26309 | Control | M | 69 | 90 |  | 0.75 | 1 | n/a |
| SD011/18 | BBN001.32848 | Control | F | 61 | 80 |  | 2.75 | 3 | 4.25 |
| SD036/17 | BBN001.30916 | Control | M | 71 | 71 |  | 1.25 | 2.75 | 6.25 |
| SD042/18 | BBN001.35138 | Control | F | 73 | 74 |  | 2.75 | 1 | 4.75 |
|  |  |  |  |  |  | **Average** | 1.75±0.935 * | 1.9±0.945* | 5.5±1.19 |
| SD037/18 | BBN001.35096 | AD | M | 72 | 103 | **MBI35 quantification**  (avg MBI35^+^ cells/fov) | 19 | 22.25 | 19 |
| SD042/17 | BBN001.31441 | AD | M | 72 | 74 |  | 16 | 14 | 10.75 |
| SD034/17 | BBN001.30883 | AD | F | 61 | 69 |  | 19.25 | 23.75 | 15 |
| SD014/19 | BBN001.35416 | AD | M | 68 | 72 |  | 18 | 20 | 15.75 |
| SD040/18 | BBN001.35110 | AD | F | 77 | 40 |  | 18 | 7.25 | 5.25 |
|  |  |  |  |  |  | **Average** | 18.05±1.279* | 17.45±6.803* | 13.15±5.305 |

Note: Comparison between respective deep, mid, and subpial GM regions of the control and AD human brain tissue. * P<0.05

Supplementary Table 2 qRT-PCR primer list

| **Gene** | **Forward (5’-3’)** | **Reverse (5’-3’)** |
| --- | --- | --- |
| **Housekeepers** | | |
| ***HPRT*** | CCTGGCGTCGTGATTAGTGA | CGAGCAAGACGTTCAGTCCT |
| ***SDHA*** | ACTGTTGCAGCACAGCTAGA | CCGCCCTTTCCAAACTTGAG |
| ***18S*** | CGGCTACCACATCCAAGGAA | GCTGGAATTACCGCGGCT |
| ***UBC*** | CGGTGAACGCCGATGATTAT | ATCTGCATTGTCAAGTGACGA |
| **Cell Markers** | | |
| ***IBA1*** | GCTGAGCTATGAGCCAAACC | TCATCCAGCCTCTCTTCCTG |
| ***GFAP*** | GAGCAGGAGGAGCGGCAC | TAGTCGTTGGCTTCGTGCTT |
| ***RBFOX3*** | AGCCTACAGCGACAGTTACG | ATGGTCCGAGAAGGAAACGG |
| **Complement** | | |
| ***CR1*** | GCGATGAAGGGTTCCGAT | GGTGTCGCATGCGTAAGA |
| **Sendai Virus clearance** | | |
| ***SeV*** | GGATCACTAGGTGATATCGAGC | ACCAGACAAGAGTTTAAGAGATATGTATC |
| ***KOS*** | ATGCACCGCTACGACGTGAGCGC | ACCTTGACAATCCTGATGTGG |
| ***Klf4*** | TTCCTGCATGCCAGAGGAGCCC | AATGTATCGAAGGTGCTCAA |
| ***c-Myc*** | TAACTGACTAGCAGGCTTGTCG | TCCACATACAGTCCTGGATGATGATG |

Supplementary Table 3 Results from screening of donors for CR1 length polymorphism, CR1 density polymorphism, and APOE status. 28 donors were screened for the density polymorphism of CR1, 15 of them for the CR1 density polymorphism and 27 for their APOE status. Representative examples of 8 donors screening are presented in Supplementary Figure 3.

| **Screening** | **Genotype** | **Number of donors** | **Total number of donors screened** |
| --- | --- | --- | --- |
| **CR1 length polymorphism** | *1/*1 | 15 | 28 |
|  | *2/*2 | 4 |  |
|  | *1/*2 | 7 |  |
|  | *3/*4 | 1 |  |
|  | *1/*3 | 1 |  |
| **CR1 density**  **polymorphism** | H/H | 10 | 15 |
|  | H/L | 5 |  |
| **APOE status** | ε3/ε3 | 21 | 27 |
|  | ε3/ε4 | 4 |  |
|  | ε2/ε4 | 1 |  |
|  | ε2/ε3 | 1 |  |

Supplementary Table 4 List of the generated iPSC lines (+KOLF2) and their CR1 length and density polymorphism genotypes and APOE status

| **iPSC lines** | ***CR1* length polymorphism** | ***CR1* density polymorphism** | **APOE**  **status** |
| --- | --- | --- | --- |
| **A** | *2/*2 | H/H | ε3/ε3 |
| **B1** | *1/*1 | H/H | ε3/ε3 |
| **B2** | *1/*1 | H/L | ε3/ε3 |
| **B3** | *1/*1 | H/H | ε3/ε4 |
| **D** | *2/*2 | n/a | ε2/ε4 |
| **E** | *1/*2 | H/L | ε3/ε4 |
| **F** | *1/*2 | H/H | ε3/ε3 |
| **KOLF2** | *3/*4 | H/H | ε3/ε3 |
